# Supplementary material for: Pyrotinib plus docetaxel as first-line treatment for HER2-positive metastatic breast cancer: the PANDORA phase II trial
Source: Nat Commun. 2023 Dec 14;14:8314. doi: 10.1038/s41467-023-44140-y (PMC10721794; doi:10.1038/s41467-023-44140-y)
Supplement: Supplementary file 1 — Supplementary Information [file 41467_2023_44140_MOESM1_ESM.pdf]

Supplementary Information

Pyrotinib plus docetaxel as first-line treatment for HER2-positive metastatic breast cancer: the PANDORA phase II trial

This PDF file includes:

Supplementary Tables 1-4

Supplementary Figures 1

Protocol

Supplementary Table 1. Treatment-related adverse events occurring in at least 5% of patients among those with or without loperamide prophylaxis.

Event, n (%)	Without loperamide prophylaxis (n=34)		With loperamide prophylaxis (n=45)	
	Any Grade	≥ Grade 3	Any Grade	≥ Grade 3
All	34 (100%)	24 (70.6%)	45 (100%)	18 (40.0%)
Diarrhea	31 (91.2%)	13 (38.2%)	18 (40.0%)	4 (8.9%)
Anemia	18 (52.9%)	1 (2.9%)	26 (57.8%)	2 (4.4%)
Leukopenia	21 (61.8%)	12 (35.3%)	20 (44.4%)	10 (22.2%)
Neutropenia	17 (50.0%)	14 (41.2%)	16 (35.6%)	9 (20.0%)
ALT/AST increased	11 (32.4%)	0	16 (35.6%)	0
Hypokalemia	18 (52.9%)	4 (11.8%)	7 (15.6%)	1 (2.2%)
Vomiting	12 (35.3%)	1 (2.9%)	13 (28.9%)	0
Hypoalbuminemia	7 (20.6%)	0	10 (22.2%)	0
Hypoproteinemia	6 (17.6%)	0	8 (17.8%)	0
Rash	6 (17.6%)	1 (2.9%)	8 (17.8%)	1 (2.2%)
Nausea	7 (20.6%)	0	6 (13.3%)	0
Thrombocytopenia	5 (14.7%)	0	6 (13.3%)	1 (2.2%)
Stomatitis	5 (14.7%)	0	5 (11.1%)	0
Urinary infection	4 (11.8%)	0	6 (13.3%)	0
Bilirubin increased	5 (14.7%)	0	5 (11.1%)	0
Creatinine increased	4 (11.8%)	0	5 (11.1%)	0
Fatigue	4 (11.8%)	1 (2.9%)	3 (6.7%)	0
Hypertriglyceridemia	5 (14.7%)	0	2 (4.4%)	0
Lymphopenia	3 (8.8%)	1 (2.9%)	3 (6.7%)	1 (2.2%)
Alkaline phosphatase increased	1 (2.9%)	0	4 (8.9%)	0

ALT, alanine transaminase; AST, aspartate transaminase.

Supplementary Table 2. Treatment-related adverse events occurring in at least 5% of patients among those with or without pegylated recombinant human granulocyte colony-stimulating factor (PEG-rhG-CSF) prophylaxis.

Event, n (%)	Without PEG-rhG-CSF prophylaxis (n=36)		With PEG-rhG-CSF prophylaxis (n=43)	
	Any Grade	≥ Grade 3	Any Grade	≥ Grade 3
All	36 (100%)	26 (72.2%)	43 (100%)	17 (39.5%)
Diarrhea	30 (83.3%)	12 (33.3%)	19 (44.2%)	5 (11.6%)
Anemia	20 (55.6%)	1 (2.8%)	24 (55.8%)	0
Leukopenia	22 (61.1%)	12 (33.3%)	19 (44.2%)	10 (23.3%)
Neutropenia	17 (47.2%)	13 (36.1%)	16 (37.2%)	10 (23.3%)
ALT/AST increased	14 (38.9%)	0	13 (30.2%)	0
Hypokalemia	16 (44.4%)	3 (8.3%)	9 (20.9%)	2 (4.7%)
Vomiting	13 (36.1%)	1 (2.8%)	12 (27.9%)	0
Hypoalbuminemia	8 (22.2%)	0	9 (20.9%)	0
Hypoproteinemia	6 (16.7%)	0	8 (18.6%)	0
Rash	7 (19.4%)	1 (2.8%)	7 (16.3%)	1 (2.3%)
Nausea	5 (13.9%)	0	8 (18.6%)	0
Thrombocytopenia	5 (13.9%)	0	6 (14.0%)	1 (2.3%)
Stomatitis	4 (11.1%)	0	6 (14.0%)	0
Urinary infection	4 (11.1%)	0	6 (14.0%)	0
Bilirubin increased	3 (8.3%)	0	7 (16.3%)	0
Creatinine increased	4 (11.1%)	0	5 (11.6%)	0
Fatigue	5 (13.9%)	1 (2.8%)	2 (4.7%)	0
Hypertriglyceridemia	4 (11.1%)	0	3 (7.0%)	0
Lymphopenia	3 (8.3%)	1 (2.8%)	3 (7.0%)	1 (2.3%)
Alkaline phosphatase increased	2 (5.6%)	0	3 (7.0%)	0

ALT, alanine transaminase; AST, aspartate transaminase.

Supplementary Table 3. The associations between biomarkers and objective response rate

Gene	ORR	No. of patients	No. of patients (WT)	No. of patients (MUT)	OR (ref=WT)	Exact P	Adjusted P
BCL2	CR	3	2 (66.7%)	1 (33.3%)	Inf	0.1071	>0.9999
	PR	25	25 (100%)	0			
BCORL1	CR	3	2 (66.7%)	1 (33.3%)	Inf	0.1071	>0.9999
	PR	25	25 (100%)	0			
FAT4	CR	3	2 (33.3%)	1 (33.3%)	10	0.2063	>0.9999
	PR	25	24 (96.0%)	1 (4.0%)			
KMT2B	CR	3	2 (33.3%)	1 (33.3%)	10	0.2063	>0.9999
	PR	25	24 (96.0%)	1 (4.0%)			
MTAP	CR	3	2 (33.3%)	1 (33.3%)	10	0.2063	>0.9999
	PR	25	24 (96.0%)	1 (4.0%)			
Gene	ORR	No. of patients	No. of patients (WT)	No. of patients (Amp)	OR (ref=WT)	Exact P	Adjusted P
MDM4	CR	3	2 (66.7%)	1 (33.3%)	2.5138	0.4594	>0.9999
	PR	25	21 (84.0%)	4 (16.0%)			
STK11	CR	3	2 (66.7%)	1 (33.3%)	5.1903	0.2979	>0.9999
	PR	25	23 (92.0%)	2 (8.0%)			

Feature	ORR	No. of patients	No. of patients (Low)	No. of patients (High)	OR (ref=Low)	Exact P	-
MSI score	CR	3	0	3 (100.0%)	Inf	0.533	-
	PR	25	9 (36.0%)	16 (64.0%)			
TMB	CR	3	2 (66.7%)	1 (33.3%)	0.4741	>0.9999	-
	PR	25	12 (48.0%)	13 (52.0%)			

In 31 patients eligible for biomarker analysis, 28 were evaluable. WT, wild type; MUT, mutation; OR, odds ratio; Inf, infinity; Amp, amplification; MSI, microsatellite instability; TMB, tumor mutation burden. Two-sided Fisher's exact test was performed in this analysis, followed by multiple testing correction via the method of Benjamini & Hochberg.

Supplementary Table 4. The association between *MYC* amplification and baseline characteristics

Variables	*MYC* amplification	*MYC* wild type	Exact P	Adjusted P	Odds ratio
Prior trastuzumab					
With prior trastuzumab (n=6)	3 (50%)	3 (50%)	0.0376	0.4131	10.1364
Without prior trastuzumab (n=25)	2 (8%)	23 (92%)			
Disease status					
Recurrent or metastatic disease (n=16)	5 (31%)	11 (69%)	0.0434	0.4772	infinity
De novo disease (n=15)	0	15 (100%)			

Two-sided Fisher's exact test was performed in this analysis, followed by multiple testing correction via the method of Benjamini & Hochberg.

Supplementary Figure 1. Mutation landscape of driver genes (n=31). ORR, objective response rate; CR, complete response; PR, partial response; SD, stable disease; NA, not available. CNV, copy number variation; SNP, single nucleotide polymorphism. Source data are provided with this paper.

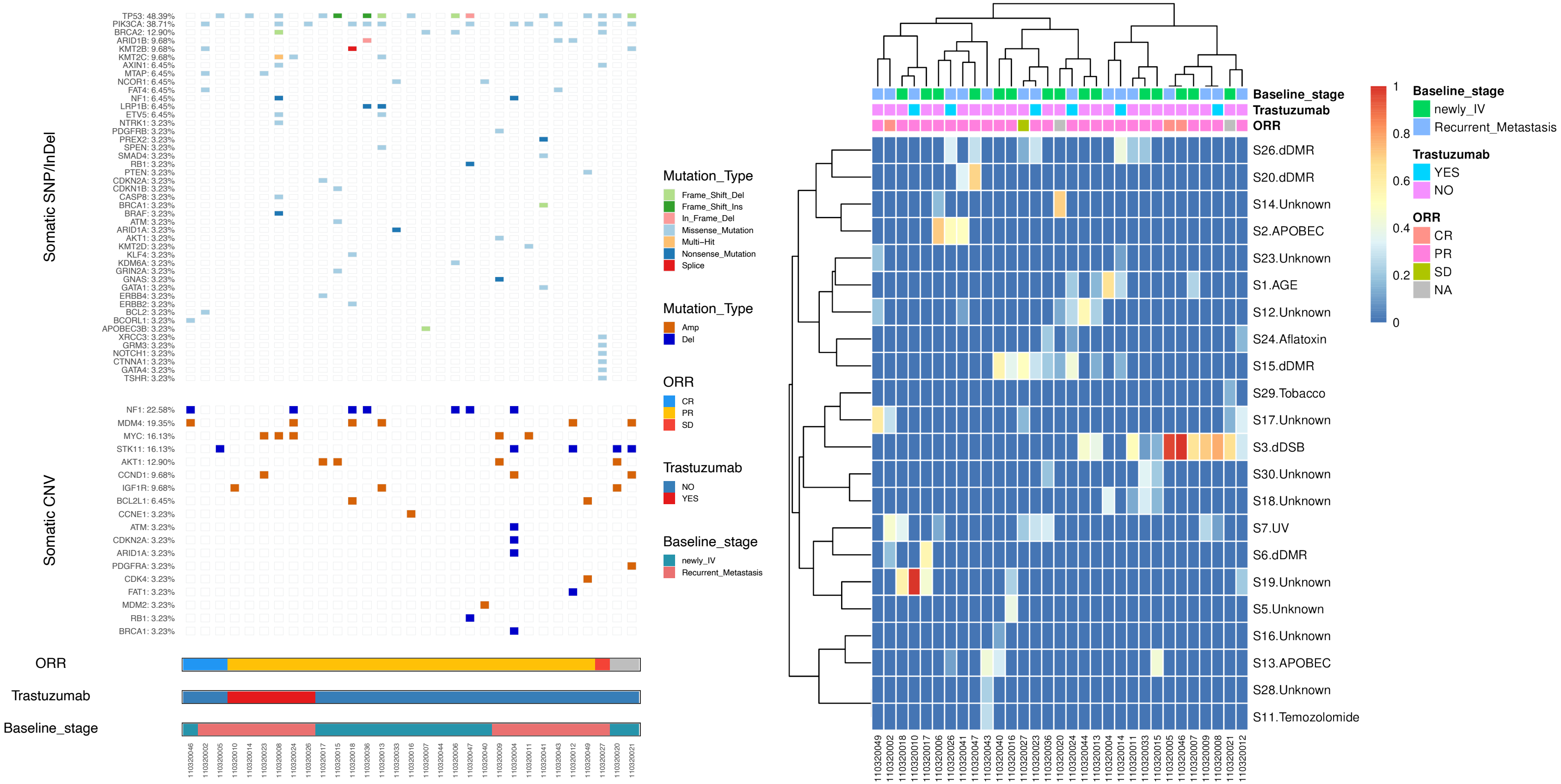

Pyrotinib in combination with docetaxel as first-line treatment for HER2-positive metastatic breast cancer (PANDORA): a single-arm, multicenter phase 2 trial

Protocol

Table of Contents

Protocol Synopsis 1
Study Flow Chart 4
List of Abbreviations 9
1 Study Background 12
1.1 Drug Name and Physicochemical Properties 14
1.2Pharmacological Types and Mechanisms of Action of Pyrotinib 14
1.3 Preclinical Pharmacodynamic Studies of Pyrotinib 15
1.4 Preclinical Toxicology Studies of Pyrotinib 15
1.5 Preclinical Pharmacokinetic Study of Pyrotinib 15
1.6 Clinical Study Progress of Pyrotinib 15
1.7 Study Rationale 16
2 Study Objectives and Study Endpoints 18
3. Study Design 18
4 Selection and Withdrawal of Subjects 19
4.1 Inclusion Criteria 19
4.2 Exclusion criteria 20
4.3 Withdrawal Criteria 21
4.4 Removal Criteria 22
4.5 Termination Criteria 22
5 Study Drugs 23
5.1 Overview of Study Drugs 23
5.2 Method of Administration 23
5.3 Management of Common Adverse Events 24
5.4 Dose Modification Scheme 27
5.5 Management, Dispensing and Return of Study Drugs 29
5.6 Compliance with Study Drug Administration 29
6 Concomitant Medications 30
6.1 Prohibited Medications During the Study 30
6.2 Medications to be Used with Caution During the Study 30
6.3 Allowed Concomitant Medications and Treatments During the Study 30
6.4 Prophylactic Antidiarrheal 31
6.5 Prophylactic Leukocyte Elevation 31
7 Study Procedures 32
7.1 Screening Period 32

7.2 Study Period 33
7.3 End of Treatment/Withdrawal from the Study 35
7.4 Follow-up Period 36
8 Efficacy Evaluation 36
8.1 Imaging 36
8.2 Primary Endpoint Measure 37
8.3 Secondary Endpoint Measures 37
9 Safety Evaluation 38
9.1 Physical Examination and Vital Signs 38
9.2 Laboratory Tests 38
9.3 Electrocardiogram 39
9.4 Cardiac Color Ultrasonography 40
9.5 Adverse Events (AEs) 40
9.6 Serious Adverse Events (SAEs) 42
9.7 Pregnancy 45
10 Retrospective Bioinformatic Analysis 45
11 Study Management 46
11.1 Ethics and Informed Consent 46
11.2 Study Drug Management 46
11.3 Amendments to the Protocol 47
11.4 Monitoring 47
11.5 Quality Control and Assurance 47
11.6 Data Management 48
11.7 Protocol Violations 49
11.8 Data Storage 49
11.9 Publication of Study Results 49
12 Data Analysis and Statistical Methods 49
12.1 Sample Size Calculation 50
12.2. Study Hypotheses 50
12.3 Data Analysis Sets 50
12.4 Statistical Analysis Plan 51
13 Dropouts 51
14 References 52

Protocol Synopsis

Study Title	Pyrotinib in combination with docetaxel as first-line treatment for HER2-positive metastatic breast cancer (PANDORA): a single-arm, multicenter phase 2 trial
Study Sponsor	***
Principle Investigator	***
Version Date	31 August 2020
Investigational Products	Pyrotinib Maleate Tablets (hereinafter referred to as pyrotinib)<br>Docetaxel Injection (hereinafter referred to as docetaxel)
Study Rationale	In the Phase 2 study of pyrotinib plus capecitabine in HER2-positive breast cancer, the primary endpoint ORR was 78.5% and the investigator-assessed PFS was 18.1 months; in the Study M77001 of trastuzumab in combination with docetaxel as the first-line treatment of HER2-positive breast cancer, ORR was 61% and PFS was 10.6 months; in Study HERNATA of trastuzumab in combination with docetaxel, ORR was 59.3% and TTP was 12.4 months.
Study Subjects	It plans to enroll 79 patients with HER2-positive metastatic breast cancer to receive the first-line therapy.
Study Objective	To explore the efficacy and safety of pyrotinib combined with docetaxel regimen in the treatment of HER2-positive metastatic breast cancer.
Endpoints	Primary Endpoint<br>Objective Response Rate (ORR)<br>Secondary Endpoints<br>Efficacy variables: progression-free survival (PFS), duration of response (DoR), clinical benefit rate (CBR), and overall survival (OS).<br>Safety variables: ECOG score, vital signs, physical examination, laboratory parameters (hematology, urinalysis and stool routine, blood biochemistry, pregnancy), ECG, echocardiography, etc.<br>Adverse events (AEs) and serious adverse events (SAEs), according to NCI-CTC AE 5.0 criteria.
Study Design	This study applies a multicenter, single-arm, open-label design, and plans to enroll 79 patients with HER2-positive metastatic breast cancer to receive the first-line treatment of pyrotinib combined with docetaxel. The primary objective is to investigate the efficacy and safety of pyrotinib combined with docetaxel as first-line treatment of HER2-positive metastatic breast cancer.<br>After signing the informed consent form, the subjects will enter the study period and receive docetaxel combined with pyrotinib until disease progression. The treatment must be discontinued in case of intolerable toxicity, withdrawal of consent or at the discretion of the investigator. Imaging assessments will be performed according to RECIST 1.1 criteria and the assessment result from the study site will be the final result.<br>Subjects who discontinue pyrotinib combined with docetaxel will enter the follow-up period (efficacy follow-up, safety follow-up, and survival follow-up):<br>Efficacy follow-up: all subjects will be followed until tumor progression, death or withdrawal of consent, whichever occurs first.<br>Safety follow-up: all subjects will be followed until he/she starts other anti-tumor drug therapy, all AEs return to Grade 0-1 or baseline, or the subject is died, whichever occurs first.<br>Survival follow-up: all subjects will be followed for survival until death, withdrawal of consent or the end of study, whichever occurs first.<br>The overall design diagram of this study is as follows:

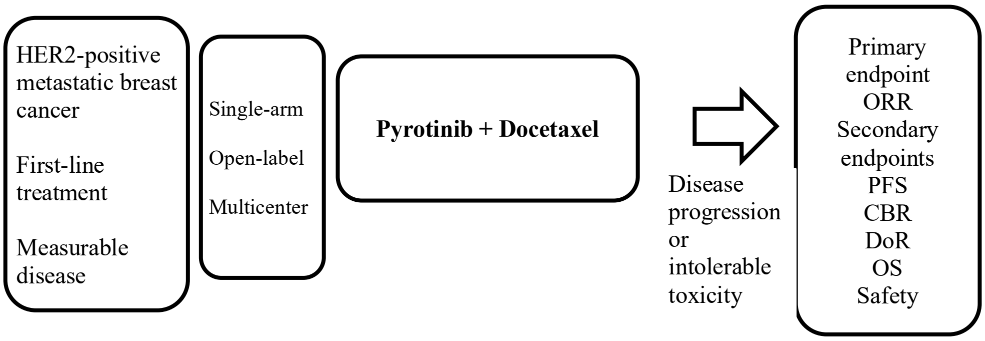

Inclusion Criteria

Subjects must meet all of the following inclusion criteria to be eligible for the study:

1. Histologically confirmed locally recurrent or metastatic breast cancer suitable for chemotherapy.
2. HER2-positive breast cancer, according to the 2018 version of ASCO-CAP criteria for HER2 positive interpretation, with immunohistochemistry (IHC) score of 3+, or 2+ and positive by in situ hybridization (ISH) test (ISH amplification rate ≥ 2.0) confirmed by pathology laboratory.
3. Recurrent or metastatic lesions have not been treated with chemotherapy, while local treatment for local symptoms, such as radiotherapy for the relief of bone pain, is allowed.
4. Patients with bilateral breast cancer who have HER2 positive metastatic lesions.
5. Age 18-70 years (inclusive).
6. ECOG score 0-1.
7. Expected survival is not less than 12 weeks.
8. Presence of at least one measurable lesion per RECIST 1.1 criteria.
9. Endocrine treatments are allowed during the recurrent or metastatic phase; prior adjuvant/neoadjuvant treatment of taxane and trastuzumab is allowed, provided that the patients have disease-free interval ≥ 12 months from the end of last adjuvant/neoadjuvant taxane therapy to tumor progression, or ≥ 6 months from the end of last adjuvant/neoadjuvant trastuzumab to tumor progression.
10. Organ function levels must meet the following requirements:

1） Bone marrow function

- ANC ≥ 1.5×10^9/L；
- PLT ≥ 75×10^9/L；
- Hb ≥ 100 g/L；

2） Liver and renal function

- TBIL≤ULN；
- ALT and AST ≤ 3 × ULN (ALT and AST ≤ 5 × ULN for patients with liver metastases);
- BUN and Cr ≤ 1.5 × ULN and creatinine clearance ≥ 50 mL/min (Cockcroft-Gault formula);

3） Cardiac color ultrasonography

LVEF ≥ 50%；

4） 12-lead ECG

QT interval ≤ 480 ms

11. Patients with known hormone receptor status.
12. Subjects who are willing to participate in this study sign the informed consent form, and have good compliance and willingness to cooperate with follow-up.

Exclusion Criteria

Subjects will be ineligible for enrollment into the study if any of the following criteria is met:

1. Patients with metastases to the central nervous system;
2. Inability to swallow, chronic diarrhea and intestinal obstruction, and multiple factors affecting drug intake and absorption;
3. Patients who have received radiotherapy, chemotherapy, surgical treatment (excluding

	local puncture) or molecular targeted therapy within 4 weeks prior to enrollment; those who have received anti-tumor endocrine therapy after screening.<br>4. Have participated in other drug clinical studies within 4 weeks prior to screening;<br>5. Prior or ongoing use of tyrosine kinase inhibitors targeting HER2 (lapatinib, lenatinib, pyrotinib, etc.)<br>6. Other malignancies within the past 5 years, excluding cured carcinoma in situ of the cervix, basal cell carcinoma of the skin, thyroid cancer, or squamous cell carcinoma of the skin.<br>7. Concurrently receive any other anti-tumor therapy.<br>8. Known history of allergy to the drug components in this protocol; history of immunodeficiency, including positive HIV test, HCV, active hepatitis B, or other acquired and congenital immunodeficiency diseases, or history of organ transplantation.<br>9. History of any cardiac disease, including: (1) arrhythmia requiring medical treatment or clinically significant; (2) myocardial infarction; (3) heart failure; (4) any other heart disease judged by the investigator to be inappropriate for participation in this study, etc.<br>10. Female patients during pregnancy and lactation, female patients of childbearing potential who have a positive pregnancy test at baseline, or female patients of childbearing potential who are unwilling to take effective contraceptive measures throughout the study.<br>11. At the discretion of the investigator, there are concomitant diseases that seriously endanger the safety of the patient or affect the completion of the study (including but not limited to serious hypertension uncontrolled by drugs, serious diabetes mellitus, active infection, etc.).<br>12. The toxicity of prior therapy did not recover to Grade 0-1 (except alopecia).<br>13. Previous confirmed history of neurological or mental disorders, including epilepsy or dementia.<br>14. Concomitant use of CYP3A4 inhibitors or inducers or medications that prolong the QT interval.<br>15. Any other circumstance that, in the opinion of the investigator, the patient is not suitable for participation in this study.
Study Progress	The first subject is estimated to be enrolled in February 2019<br>The last subject is estimated to be enrolled in September 2020

Study Flow Chart

Item / Time	Screening Period	Study Period								Follow-up Period		
		C1			C2			C3+	Withdrawal from study	Safety	Efficacy [19]	Survival [20]
	D-28 to D-1	D7±3	D14±3	D21±3	D7±3	D14±3	D21±3	D21±3				
Signing the ICF	×											
Demographic data [1]	×											
Medical history inquiry [2]	×											
ECOG score	×	At the end of every 2 cycles within 36 cycles, and at the end of every 4 cycles after 37 cycles							×			
Vital signs [3]	×	×	×	×	×	×	×	×	×			
Physical examination [4]	×	At the end of every 2 cycles within 36 cycles, and at the end of every 4 cycles after 37 cycles							×			
Hematology [5]	×	×	×	×	×	×	×	×	×			
Urinalysis [6]	×	Test if necessary							×			
Stool routine [7]	×			×			×	×	×			
Blood chemistry [8]	×	×	×	×	×	×	×	×	×			
Coagulation test [9]	×											
Infectious disease screening [10]	×											
Pregnancy test [11]	×								×			
12-Lead ECG [12]	×	×	×	×	×	×	×	×	×			
Cardiac color ultrasonography [13]	×	Every 2 cycles							×			
Tumor imaging [14]	×	Every 6 weeks							×		×	
Pyrotinib [15]		Once daily, continuous dosing										
Docetaxel [15]		Docetaxel once every 21 days										

Drug return/dispensing [16]				×			×	×	×			
Concomitant medications/treatments [17]	×											
AE observation records [18]	×											
Time to progression/death											×	×
Subsequent tumor therapy [21]										×	×	×

Note: the following tests should be completed according to the time windows listed in the study procedures. In case of legal holidays, the tests can be advanced accordingly and the reasons for the out-of-window should be recorded in the eCRF. C1D1 is the date of the first dose of docetaxel, and the date of D1 of each cycle is calculated as 21 days after the date of docetaxel administration in the previous cycle. If the dose of docetaxel is delayed as specified in the protocol, the postponement of the visit date is not a protocol deviation; if the docetaxel administration ends, the visit date thereafter is calculated according to the date of the last docetaxel dose. The visit date is fixed and does not change according to the actual visit date. Visit at the end of Week 1, 2 and 3 of Cycle 1 (D7 ± 3, D14 ± 3, D21 ± 3) and at the end of Week 1, 2 and 3 of Cycle 2 (D7 ± 3, D14 ± 3, D21 ± 3); thereafter once at the end of each cycle (D21 ± 3) until Cycle 12; once at the end of every 2 cycles (D21 ± 3) for Cycle 13 to 36; and once at the end of every 4 cycles (D21 ± 3) after Cycle 37. The investigator may add tests or increase the frequency of visits based on the subject's clinical condition.

1. Demographic data (initials, gender, ethnicity, marital status, date of birth, height, weight, and body surface area calculated accordingly);
2. Medical history inquiry: including past medical history and treatment history (clinical/pathological diagnosis, time of diagnosis, clinical/pathological stage, HER2/ER/PR/expression; whether the patients have received surgery, neoadjuvant therapy, adjuvant therapy, or radiotherapy; progress time and rationale for diagnosis of progression; drug name, usage, time and efficacy of each line of advanced systemic therapy; whether applying other treatment, such as surgery), smoking and alcohol consumption history (frequency, amount, duration), drug allergy history (drug name, allergic symptoms), prior or concomitant diseases/symptoms (disease/symptom name, concomitant medication name, dose, usage, outcome), and stool habits (number of times);
3. Vital signs: including body temperature, blood pressure, respiratory rate, and pulse; once at screening, once at the end of Week 1, 2 and 3 of Cycle 1 (D7 ± 3, D14 ± 3, D21 ± 3), once at Week 1, 2 and 3 of Cycle 2 (D7 ± 3, D14 ± 3, D21 ± 3), thereafter once every cycle until Cycle 12 (D21 ± 3), every 2 cycles in Cycle 13 to 36 (D42 ± 3), every 4 cycles after Cycle 37 (D84 ± 3), and once at the end of treatment/withdrawal (if not performed within 7 days before);
4. Physical examination: including general condition, skin and mucosa, lymph nodes, head and neck, chest, abdomen, muscles and bones, neuroreflexes, respiratory system, cardiovascular system, genitourinary system, mental status, etc.; once at screening and at the end of every 2 cycles until Cycle 36, every 4 cycles after 37 cycles, and once at the end of treatment/withdrawal (if not performed within 7 days before);

5. Hematology: including absolute counts of WBC, ANC, LC, RBC, Hb and PLT; once at Week 1, 2 and 3 of Cycle 1 (D7 ± 3, D14 ± 3, D21 ± 3), once at Week 1, 2 and 3 of Cycle 2 (D7 ± 3, D14 ± 3, D21 ± 3), thereafter once every cycle until Cycle 12 (D21 ± 3), every 2 cycles in Cycle 13 to 36 (D42 ± 3), every 4 cycles after Cycle 37 (D84 ± 3), and once at the end of treatment/withdrawal (if not performed within 7 days before) (test in another hospital may be acceptable);
6. Urinalysis: including urine protein, glucose, and occult blood, if urinalysis shows urine protein ++ or above, please add the 24-h urine protein quantification test; once at screening, at the end of treatment/withdrawal (if not performed within 7 days before), and at other times as judged by the investigator;
7. Stool routine: including stool appearance and fecal occult blood; at the visit every cycle from screening to Cycle 12 (D21 ± 3), every 2 cycles from Cycle 13 to 36 (D42 ± 3), every 4 cycles after Cycle 37 (D84 ± 3), and at the end of treatment/withdrawal (if not performed within 7 days before) (test in another hospital may be acceptable);
8. Blood chemistry: including glucose, TP, ALT, AST, ALP, γ-GT, ALB, TBIL, DBIL, IBIL, TG, CHOL, UA, BUN, Cr, K^+, Na^+, CL^-, and Ca^{2+}; once at Week 1, 2 and 3 of Cycle 1 (D7 ± 3, D14 ± 3, D21 ± 3), once at Week 1, 2 and 3 of Cycle 2 (D7 ± 3, D14 ± 3, D21 ± 3), thereafter once every cycle until Cycle 12 (D21 ± 3), every 2 cycles in Cycle 13 to 36 (D42 ± 3), every 4 cycles after Cycle 37 (D84 ± 3), and once at the end of treatment/withdrawal (if not performed within 7 days before); myocardial zymogram may be performed if necessary, at the discretion of the investigator according to the subject's condition;
9. Coagulation function: including prothrombin time (PT), activated partial thromboplastin time (ATTP), thrombin time (TT), and national normalized ratio (INR); once at the screening period;
10. Infectious disease screening: including hepatitis B five items, HIV antibody, and HCV antibody testing; once at the screening period;
11. Pregnancy test: female subjects of childbearing potential need to undergo blood HCG test to exclude pregnancy at screening; once at screening period and once at the end of treatment/withdrawal;
12. 12-lead ECG: 3 times at the baseline, 10 minutes apart is recommended; once at Week 1, 2 and 3 of Cycle 1 (D7 ± 3, D14 ± 3, D21 ± 3), once at Week 1, 2 and 3 of Cycle 2 (D7 ± 3, D14 ± 3, D21 ± 3), thereafter once every cycle until Cycle 12 (D21 ± 3), every 2 cycles in Cycle 13 to 36 (D42 ± 3), and every 4 cycles after Cycle 37 (D84 ± 3). During the study, if the QTc interval increases >30 msec from baseline, or the absolute value of QTc interval ≥ 480 msec occurs in any specified ECG measurement, two additional ECGs (at least 10 minutes apart is recommended) are required (examination in another hospital is acceptable).
13. Cardiac color ultrasonography: during the screening period; if there are symptoms such as chest pain and palpitations during the study, additional examinations may be performed as appropriate; thereafter, once at the end of every 2 cycles, and once at the end of treatment/withdrawal (if not performed within the previous 4 weeks);
14. Tumor imaging: including chest and abdomen examination. Craniocerebral examination is required at baseline to exclude brain metastases. Neck and pelvic examination, bone scan, etc. may be added as clinically indicated; enhanced CT, PET-CT or MRI may be used. For patients with bone metastases at baseline, bone scan should be performed at least once every 6 months (approximately 180 days), and bone scans are not required routinely in general patients unless bone metastases

are suspected. The investigator may add scan sites in tumor assessments at or after baseline as clinically indicated. Reports within 28 days prior to screening may be used. Tumor imaging time points during the dosing period will be determined after the start of study treatment, regardless of the time of dose interruption due to toxicity during this period. The allowed time window for tumor imaging is ± 7 days, and the specific assessment time points are as follows:

- ✓ Every 6 weeks (regardless of treatment delay);
- ✓ In the event of radiologically confirmed PD, the subject will discontinue the study treatment and enter the follow-up period. No other anti-tumor therapy can be performed prior to PD;

15. Dosing regimen:

Pyrotinib: 400 mg once daily orally within 30 minutes of breakfast for 21 consecutive days in a cycle until disease progression or patient intolerance.

Docetaxel: 75 mg/m^2 administered on D1 of each cycle of 21 days, for 6 cycles, by intravenous infusion (allowable range of routine use is ± 10%), and docetaxel could only be discontinued in case of disease progression or intolerable toxicity before completion of 6 cycles. After the end of the 6th cycle, the patient and doctor will carefully consider and decide whether to discontinue docetaxel.

• The above medications may be adjusted based on the adverse reactions of the subjects according to the protocol. Subjects will continue the treatment until disease progression, intolerable toxicity, withdrawal of informed consent, or discontinuation at the discretion of the investigator.

16. Drug return/dispensing: drug return and dispensing will be recorded at the end of each cycle.
17. Concomitant medications/treatments: concomitant medications during the screening period and during the study should be recorded; once the subject interrupts the study treatment, only concomitant medications and therapies for new or unresolved AEs related to the study treatment should be recorded.
18. AE observation records: monitor AEs from the day of signing the informed consent form until at least 28 days after the last dose; record in detail the AEs, concomitant medications/treatments and unscheduled examinations during this period.
19. Efficacy follow-up: subjects who withdraw from the study due to non-PD and non-death causes should be followed for efficacy. Tumor imaging assessment continues at the time points specified in the protocol (every 6 weeks within 20 cycles and every 12 weeks after 21 cycles) from the last tumor imaging assessment during the study until PD, initiation of other anti-tumor drugs, or death, whichever comes first. Each follow-up time, tumor imaging assessment results and other anti-tumor treatment information during this period will be recorded in detail.
20. Survival follow-up (OS data collection): all survived subjects who complete the safety follow-up and efficacy follow-up (whichever is later) need to undergo survival follow-up. Survival information (date and cause of death) and information after the study treatment (including the received treatment) will be collected by telephone or clinical follow-up of the subject, his/her family or local doctor at least once every 12 weeks (± 7 days) from the date of completion of the safety follow-up visit and efficacy follow-up visit (whichever is later) until death, subject is lost to follow-up, or the end of OS data collection, whichever occurs first. The details of each

survival follow-up visit should be recorded in detail and entered in the corresponding eCRF.

21. Tumor treatment: whether the subject uses other tumor therapy should be recorded from the time of withdrawal to the end of survival follow-up; concomitant medications due to other diseases need not be recorded.

List of Abbreviations

Abbreviations	Chinese
ADL (Activities of Daily Living)	日常生活活动
AE (adverse event)	不良事件
AKP/ALP (Alkaline Phosphatase)	碱性磷酸酶
ALB (albumin)	白蛋白
ALT (alanine aminotransferase)	丙氨酸氨基转移酶
ANC (absolute neutrophil count)	中性粒细胞计数
AST (aspartate aminotransferase)	天门冬氨酸氨基转移酶
AUC (area under concentration time curve)	药-时曲线下面积
BMI (Body Mass Index)	身体质量指数
BUN (blood urea nitrogen)	尿素氮
NMAP (National Medical Products Administration)	中国食品药品监督管理局
CHOL (cholestenone)	胆固醇
CI（Confidence Interval）	可信区间
CL/F	表观口服清除率
Cmax (peak concentration)	峰浓度
Cr (creatinine)	肌酐
CR (Complete Response)	完全缓解
CRF (case report form)	病例报告表
DBIL (direct bilirubin)	直接胆红素
dL (deciliter)	分升
DLT (dose limited toxicity)	剂量限制毒性
DPD (dihydropyrimidine Dehydrogenase)	二氢嘧啶脱氢酶
EC (ethics committee)	伦理委员会
ECOG (Eastern Cooperative Oncology Group)	美国东部肿瘤协作组
EDC (electronic data collection)	电子数据采集系统
EGF (Epidermal Growth Factor）	表皮生长因子
EGFR (epidermal growth factor receptor)	表皮生长因子受体
ER (estrogen receptor)	雌激素受体
FAS (Full analysis set)	全分析集
FISH (fluorescence in situ hybridization)	荧光原位杂交技术
g (gram)	克/离心力
GCP (good clinical practice)	临床试验规范
h (hour)	小时
Hb (hemoglobin)	血红蛋白
HCV (Hepatitis C Virus)	丙型肝炎病毒
HER2 (human epidermal growth factor receptor 2)	人表皮生长因子受体 2
HIV (Human Immunodeficiency Virus)	人类免疫缺陷病毒
HR (heart rate)	心率

IBIL (indirect bilirubin)	间接胆红素
IC_{50} (50% Inhibition Concentration)	半数抑制浓度
ITT (intend to treat)	治疗意图
IU (international unit)	国际单位
IV (intravenous)	静脉注射（滴注）
kg (kilogram)	千克
L（Liter）	升
LC (lymphocyte cont)	淋巴细胞计数
LDH (lactate dehydrogenase)	乳酸脱氢酶
LVEF（Left Ventricular Ejection Fraction）	左心室射血分数
m (meter)	米
min (minute)	分钟
mg (milliram)	毫克
ml (milliliter)	毫升
mm (millimeter)	毫米
ms (Millisecond)	毫秒
MTD (maximum torlerate dose)	最大耐受药物剂量
NCCN (National Comprehensive Cancer Network)	美国国立综合癌症网络
NCI-CTC (national cancer institute common terminology criteria)	国立癌症研究所通用毒性标准
NE	无法评估
ORR (Objective Response Rate)	客观有效率
PD (Pharmacodynamics)	药物效应动力学
PFS (Progression Free Survival)	无进展生存期
PK (pharmacokinetics)	药代动力学
PLT (platelet)	血小板
PPS (Per Protocol Set)	符合方案分析集
PR（Partial Response）	部分缓解
PR（Progesterone receptor）	孕激素受体
R	蓄积因子
RBC (red blood cell)	红细胞计数
RTK (Receptor Tyrosine Kinase)	受体酪氨酸激酶
SAE (serious adverse event)	严重不良事件
SAP (statistical analysis plan)	统计分析计划
SBP (systolic blood pressure)	收缩期血压
SD (Stable Disease)	疾病稳定
SS (Safety Set)	安全性分析集
T1/2	半衰期
TBIL (total bilirubin)	总胆红素
TG (triglyceride)	甘油三酯

Tmax (peak time)	达峰时间
TP (plasma total protein)	总蛋白
TTP (Time To Progression)	疾病进展时间
μmol（micromole）	微摩尔
ULN (upper normal limit)	正常值上限
VEGF（Vascular Endothelial Growth Factor）	血管内皮生长因子
WBC (white blood cell)	白细胞计数

1 Study Background

Receptor tyrosine kinases (RTKs) are a class of transmembrane proteins involved in growth factor signaling, including an extracellular domain containing ligand binding sites, a single transmembrane hydrophobic α-helical region, and an intracellular domain containing receptor tyrosine kinase (RTK) activity. The occurrence and development of many tumors are closely related to the abnormal expression of tyrosine kinases. The available data show that more than 50% of proto-oncogenes and oncogene products have tyrosine kinase activity, and their abnormal expression will lead to the disturbance of cell proliferation regulation, thereby causing tumorigenesis, which is closely related to tumor invasion, metastasis and chemotherapy resistance. Receptor tyrosine kinases include epidermal growth factor (EGF) receptor, fibroblast growth factor (FGF) receptor, vascular endothelial growth factor (VEGF) receptor, etc.

Table 1 Drugs Targeting HER2 Molecules Currently Marketed or to Be Marketed Soon

	Drug Name	Development Company	Marketed in USA	Indications	Marketed in China
Macromolecular drugs	Trastuzumab	Roche	1998 Herceptin	HER2-positive breast cancer; HER2-positive metastatic gastric cancer and gastroesophageal junction adenocarcinoma	2002 赫赛汀
	Pertuzumab	Roche	2012 Perjeta	Preoperative neoadjuvant therapy for HER2-positive early or locally advanced breast cancer; adjuvant therapy for high-risk populations with HER2-positive breast cancer; and first-line treatment of HER2-positive metastatic breast cancer in combination with trastuzumab and docetaxel	2019 帕捷特
	Inetetamab Cipterbin®	Sunshine Guojian	2020	Treatment of HER2-positive metastatic breast cancer in combination with chemotherapy	2020 赛普汀
Antibody-drug conjugates	T-DM1	Roche	2013 Kadcyla	Monotherapy for HER2-positive metastatic breast cancer previously treated with trastuzumab in combination with taxane chemotherapy	2020 赫赛莱
First-generation small molecule inhibitors	Lapatinib	GSK	2007 Tykerb	Combined with capecitabine in treatment of patients with HER2-positive advanced breast cancer previously treated with trastuzumab in combination with taxane and anthracycline chemotherapy; and combined with letrozole in treatment of postmenopausal patients with HR-positive, HER-positive metastatic breast cancer	2013 泰立沙
Second-generation small	Neratinib	Puma	2017 Nerlynx	For patients with breast cancer who have completed standard adjuvant	2020 贺俪安

molecule inhibitors				treatment of trastuzumab (赫赛汀, Herceptin) and have not progressed but have high risk factors	
Small molecule inhibitors independently developed by Hengrui	Pyrotinib	Jiangsu Hengrui Medicine	-	Combined with capecitabine in the first- and second-line treatment of Her2-positive recurrent/metastatic breast cancer	2018 艾瑞妮

The EGFR family consists of four members, EGFR/HER1/erbB-1, HER2/erbB-2, HER3/erbB-3, and HER4/erbB-4. In the 1980s, scientists found that HER2 amplification and overexpression were associated with a more aggressive tumor phenotype. So far, no ligands have been found to bind to HER2 receptors. HER2 molecules mainly form heterodimers with other receptors of the EGFR family to further activate MAPK, JAK, PI3K, STAT3 and other pathways to promote the occurrence and development of tumor diseases. HER2 gene expression is low in normal epithelial cells while it is amplified/overexpressed in more than 30% of human tumors, including breast cancer, gastric cancer, lung cancer, etc. HER2 has received extensive attention as a target of drug action. With the standardization of HER2 amplification testing, targeted therapy for HER2-positive patients has become a hotspot in basic and clinical studies [1]. Several HER2-targeted drugs are currently marketed worldwide (see Table 1 for details).

Approximately 20% -30% of Chinese breast cancer patients have HER2 gene amplification/overexpression. The HER2-targeted drugs currently marketed in China included trastuzumab, lapatinib, and pyrotinib. According to the Chinese Society of Clinical Oncology (CSCO) Guidelines for the Diagnosis and Treatment of Breast Cancer 2018 V1, trastuzumab combined with taxane is the preferred first-line treatment of HER2-positive advanced recurrent and metastatic breast cancer in China. The Study M77001 results showed that trastuzumab combined with docetaxel as first-line treatment of HER2-positive advanced breast cancer was superior to docetaxel alone, with median TTP prolongation of 5.6 months and median OS prolongation of 8.5 months. Cardiac function assessment before treatment and cardiac function testing during treatment effectively reduced the incidence of cardiac events, indicating that trastuzumab combined with docetaxel significantly improved the survival of patients with HER2-positive advanced breast cancer [2]. The Study HERNATA results showed similar efficacy and less toxicity of vinorelbine plus trastuzumab compared with docetaxel plus trastuzumab in the first-line treatment of metastatic or locally advanced HER2-positive breast cancer, and vinorelbine plus trastuzumab may be a better first-line option [3]. When disease progression occurred after trastuzumab treatment, the following treatment strategies could be selected: 1) lapatinib in combination with capecitabine; 2) trastuzumab in combination with other chemotherapeutic agents; 3) trastuzumab in combination with lapatinib; and 4) T-DM1

monotherapy. Currently, the Phase 2 study of pyrotinib plus capecitabine has shown median PFS of 18.1 months, with few adverse reactions, and most adverse reactions are reversible. The 2020 ASCO General Assembly published the Phase 3 PHOEBE study of pyrotinib in combination with capecitabine in HER2-positive metastatic breast cancer. The study results showed median PFS of 12.5 months compared with 6.8 months in the control group, and the patients' disease-free survival were significantly prolonged with good safety. The Study PHOEBE further validated the Phase 2 study of pyrotinib, providing a new treatment option for HER2-positive metastatic breast cancer. At present, all authoritative guidelines in China and abroad emphasize that trastuzumab combined with pertuzumab combined with taxane is the first-line treatment regimen for Her2-positive advanced breast cancer, and this clinical study protocol is also intended to investigate the efficacy and safety of pyrotinib combined with docetaxel in the first-line treatment of Her2-positive advanced breast cancer.

1.1 Drug Name and Physicochemical Properties

[Generic Name] 吡咯替尼

[Chinese Pinyin] Biluotini

[English Name] Pyrotinib

[Chinese Chemical Name]（R,E）-N-（4-（3-氯-4-（吡啶-2-基甲氧基）苯基氨基）-3-氰基-7-乙氧基喹啉-6-基）-3-（1-甲基吡咯烷基-2-基）丙烯酰胺，马来酸盐(1:2)

[English Chemical Name](R,E)-N-(4-(3-chloro-4-(pyridin-2-ylmethoxy)phenylamino)-3-cyano-7-ethoxyquinolin-6-yl)-3-(1-methylpyrrolidin-2-yl)acrylamide, maleic acid salt.

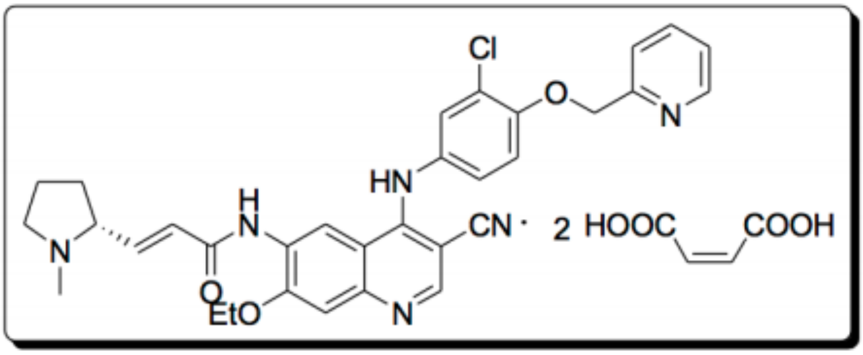

Figure 1 Chemical Structure Formula of Pyrotinib

[Molecular Formula] C40H39ClN6O11, the molecular structure is shown in Figure 1.

[Molecular Weight] 814.23

1.2Pharmacological Types and Mechanisms of Action of Pyrotinib

Pyrotinib is an irreversible inhibitor of small molecule receptor tyrosine kinases that inhibit epidermal growth factor receptor (EGFR) and human epidermal factor receptor 2 (HER2).

Pyrotinib covalently binds to ATP binding sites in the kinase region of intracellular EGFR and HER2, prevents the formation of homodimers and heterodimers of EGFR and HER2 in tumor cells, inhibits their autophosphorylation, and blocks activation of downstream signaling pathways, thereby inhibiting tumor cell growth.

1.3 Preclinical Pharmacodynamic Studies of Pyrotinib

Pyrotinib significantly inhibited EGFR and HER2 at the molecular level with 50% inhibitory concentrations (IC_{50}) of 5.6 nM and 8.1 nM, respectively. It had a strong inhibitory effect on the proliferation of HER2-overexpressing tumor cells with IC_{50} between 1 and 43 nM. It significantly inhibited the phosphorylation of EGFR and HER2 and inhibited the activation of downstream signals ERK1/2 and Akt, and the inhibitory activity was irreversible. Pyrotinib significantly caused BT474 cells to be arrested in the G1 phase of the cell cycle. Pyrotinib significantly inhibited the growth of HER2-overexpressing tumor models such as SK-OV-3, Calu-3 and BT-474 in a significant dose-dependent manner, and caused some tumor shrinkage. Its in vivo and in vitro anti-tumor effects were superior to or equivalent to the active comparator HKI-272, as detailed in Table 2; therefore, preclinical data support the further clinical studies of pyrotinib.

Table 2 Inhibition of Tyrosine Kinases by Pyrotinib

Kinase Name	IC_{50} (nM, mea±SD)	
	Pyrotinib	Neratinib (HKI-272)
HER2	8.1±2.3	6.8±0.9
EGFR1	5.6±3.9	4.2±0.9
c-Src	790.3±190.7	1159.1±1036.5
KDR	>3,000	>3,000
c-Kit	>3,000	>3,000
PDGFRβ	>3,000	>3,000
C-Met	>3,000	>3,000

1.4 Preclinical Toxicology Studies of Pyrotinib

Preclinical toxicology studies of pyrotinib include safety pharmacology, acute toxicity, long-term toxicity, and genotoxicology studies, and the study results are described in detail in the Investigator's Brochure for Pyrotinib Maleate.

1.5 Preclinical Pharmacokinetic Study of Pyrotinib

Multiple studies including absorption study in rats, in vivo pharmacokinetic study in Beagle dogs, plasma protein binding assay, tissue distribution study in rats and in vitro metabolism study have been completed, as detailed in the Investigator's Brochure of Pyrotinib Maleate.

1.6 Clinical Study Progress of Pyrotinib

Jiangsu Hengrui Pharmaceuticals Co., Ltd. was approved by the National Medical Products Administration (NMPA [formerly CFDA]) in May 2012 to start the Phase 1 clinical

development of pyrotinib; in November 2015, Hengrui obtained the approval from CFDA for conducting the Phase 2/3 clinical study. On 30 June 2015, the Phase 1 clinical development of pyrotinib was initiated in the United States (US Investigational New Drug [IND] Application Number: 126107). Currently, Jiangsu Hengrui Pharmaceuticals Co., Ltd. has conducted a total of 12 clinical studies of pyrotinib in China: 7 Phase I clinical studies, including 4 studies in healthy subjects, 2 studies in subjects with HER2-positive metastatic breast cancer (mBC) (Study BLTN-Ib, Study BLTN-Ic), and 1 study in subjects with advanced gastric cancer (Study BLTN-Id); 1 Phase 1/2 clinical study in subjects with mBC (HR-BLTN-I/II-mBC); 1 ongoing Phase 2 clinical study in HER2-mutant non-small cell lung cancer (NSCLC) (HR-BLTN-II-NSCLC study) and 3 ongoing Phase 3 clinical studies in subjects with mBC (Study HR-BLTN-III-MBC-A, HR-BLTN-III-MBC-B and HR-BLTN-III-MBC-C). Currently, there is 1 Phase 1 clinical study ongoing in the United States in subjects with HER2-positive solid tumors and HER2-mutant NSCLC who have progressed on prior HER2-targeted therapy.

According to the published results of current studies, 38 subjects were exposed to pyrotinib in the Phase 1 studies, 65 subjects were exposed to pyrotinib in the Phase 2 studies, 186 subjects were exposed to pyrotinib in the Phase 3 PHENIX study, and 134 subjects were exposed to pyrotinib in the Phase 3 PHOEBE study. In addition, the foreign subjects who received pyrotinib included 31 subjects with HER2-mutant NSCLC and 31 subjects with other solid tumors.

1.7 Study Rationale

Breast cancer is the most common malignant tumor in women in China, and it has become the fifth leading cause of cancer death in women, which seriously threatens women's life and health. Its incidence of cancer in Chinese women is the highest and is on the rise, and the age of onset is younger than that in western countries. According to the 2017 Chinese Annual Report of Tumor Grade, breast cancer has the highest incidence in female malignant tumors in China, with about 279,000 new cases per year, and is increasing at a rate of about 2% per year [4]. HER2 molecule is an independent factor for poor prognosis of breast cancer, and about 20% to 30% of Chinese breast cancer patients have HER2 gene amplification/overexpression. According to the NCCN guidelines and Chinese diagnosis and treatment practice, treatment regimens with HER2-targeted agents in combination with chemotherapy should be the preferred recommended regimen for patients with advanced breast cancer at the time of disease progression. Although trastuzumab and lapatinib have been marketed, and trastuzumab has been included in Chinese national health insurance in 2017, it is inevitable that new HER2 agents are urgently needed for the further treatment in the setting of HER2-positive breast cancer progression or patient intolerance, even if the above-mentioned HER2-targeted drugs

are available and the treatment is effective. Pyrotinib provides a new option for the first- and second-line treatment of HER2-positive recurrent and metastatic breast cancer.

Pyrotinib is a novel irreversible small molecule inhibitor of tyrosine kinase EGFR and HER2 independently developed by Jiangsu Hengrui Pharmaceuticals Co., Ltd. In the past 2 months of marketing, pyrotinib has been found to be safe and well tolerated in HER2-positive breast cancer patients in clinical use, and AEs were predictable and clinically manageable. Similar to EGFR/HER2-targeted drugs of the same class, it mainly had adverse reactions related to common pharmacological mechanisms such as diarrhea and rash. The vast majority of AEs were mild to moderate and clinically manageable. None of the AEs led to dose interruption, dose modification or discontinuation. No clinically significant abnormalities or abnormal changes in laboratory tests, vital signs, physical examinations or electrocardiograms (including QTc) parameters were found, except for AEs that have been reported. In terms of efficacy, pyrotinib or pyrotinib in combination with chemotherapy showed certain anti-tumor effects in advanced breast cancer and gastric cancer. Especially in advanced breast cancer, the results of the Phase 2 study of pyrotinib plus capecitabine showed that pyrotinib had a highly effective, rapid and sustained anti-tumor effect, and the efficacy was significantly superior to that of the lapatinib plus capecitabine control group. Currently, two Phase 3 studies of pyrotinib (Study PHENIX was published by ASCO in 2019 and Study PHOEBE was published by ASCO in 2020) have further demonstrated significant efficacy of pyrotinib in the treatment of HER2-positive breast cancer. At present, all first-line treatment regimens for advanced HER2 breast cancer in China and abroad are taxane combined with trastuzumab and pertuzumab, which shows the status of taxane in the first-line treatment of breast cancer. Since pyrotinib combined with capecitabine has achieved significant anti-tumor effects, with the median PFS of 18.1 months and ORR of 78.5%, so the efficacy of pyrotinib combined with taxane is worthy of expectation. This clinical study protocol will further investigate the efficacy and safety of pyrotinib combined with docetaxel in the first-line treatment of Her2-positive advanced breast cancer.

2 Study Objectives and Study Endpoints

Table 3 Study Objectives and Study Endpoints

Study Objective	Study Endpoints
Primary Objective	
To assess the efficacy of pyrotinib combined with docetaxel as first-line treatment of HER2-positive metastatic breast cancer	Objective response rate (ORR) as assessed by the investigator per RECIST 1.1 criteria
Secondary Objectives	
To assess the efficacy of pyrotinib combined with docetaxel as first-line treatment of HER2-positive metastatic breast cancer	PFS, DoR, CBR, and OS as assessed by the investigator (per RECIST 1.1 criteria).
To assess the safety of pyrotinib combined with docetaxel as first-line treatment of HER2-positive metastatic breast cancer	ECOG score, vital signs, physical examination, laboratory parameters (hematology, urinalysis and stool routine, blood biochemistry, pregnancy test and virological screening), ECG, echocardiography, adverse events (AEs) and serious adverse events (SAEs) according to NCI-CTCAE 5.0 criteria

3. Study Design

This study applies a multicenter, single-arm, open-label design, and plans to enroll 79 patients with HER2-positive advanced breast cancer to receive the treatment of pyrotinib combined with docetaxel. The primary objective is to investigate the efficacy and safety of pyrotinib combined with docetaxel as first-line treatment of HER2-positive breast cancer.

Subjects will enter the study period after signing the informed consent form and receive docetaxel 75 mg/m^2 on D1 of each cycle of 21 days, for 6 cycles, by intravenous infusion (allowable range of routine use is ± 10%), and docetaxel could only be discontinued in case of disease progression or intolerable toxicity before completion of 6 cycles. After the end of the 6th cycle, the patient and doctor will carefully consider and decide whether to discontinue docetaxel. The test product (pyrotinib) will be started at 400 mg once daily orally within 30 minutes of breakfast for 21 consecutive days in a cycle, and it will be continued until disease progression. The treatment must be discontinued in case of intolerable toxicity, withdrawal of consent or at the discretion of the investigator. Imaging assessments will be performed according to RECIST 1.1 criteria and the assessment result from the study site will be the final result.

Subjects who discontinue pyrotinib and docetaxel will enter the follow-up period: 1) the safety follow-up will continue until 30 days after the last dose; 2) subjects who discontinue the treatment due to non-disease progression (PD) or non-death reasons should be followed for efficacy until disease progression, start of other anti-tumor drug therapy, or death, whichever occurs first; and 3) all subjects will be followed for survival until death or the end of study,

whichever occurs first.

The overall design diagram of this study is as follows:

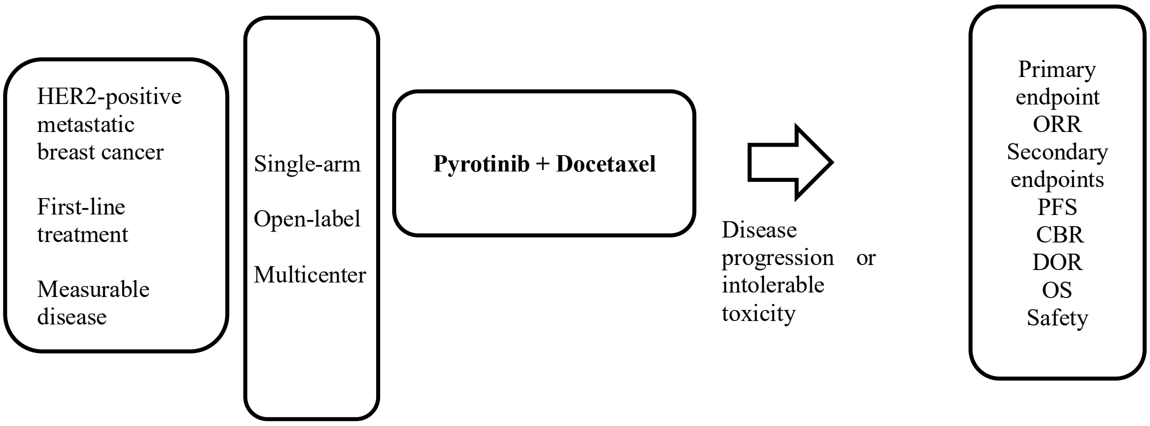

Figure 2 Overall Study Design Diagram

4 Selection and Withdrawal of Subjects

4.1 Inclusion Criteria Subjects must meet all of the following inclusion criteria to be eligible for the study:

1. Histologically confirmed locally recurrent or metastatic breast cancer suitable for chemotherapy.
2. HER2-positive breast cancer, according to the 2018 version of ASCO-CAP criteria for HER2 positive interpretation, with immunohistochemistry (IHC) score of 3+, or 2+ and positive by in situ hybridization (ISH) test (ISH amplification rate ≥ 2.0) confirmed by pathology laboratory.
3. Recurrent or metastatic lesions have not been treated with chemotherapy, while local treatment for local symptoms, such as radiotherapy for the relief of bone pain, is allowed.
4. Patients with bilateral breast cancer who have HER2 positive metastatic lesions.
5. Age 18-70 years (inclusive).
6. ECOG score 0-1.
7. Expected survival is not less than 12 weeks.
8. Presence of at least one measurable lesion per RECIST 1.1 criteria.
9. Endocrine treatments are allowed during the recurrent or metastatic phase; prior adjuvant/neoadjuvant treatment of taxane and trastuzumab is allowed, provided that the patients have disease-free interval ≥ 12

months from the end of last adjuvant/neoadjuvant taxane therapy to tumor progression, or ≥ 6 months from the end of last adjuvant/neoadjuvant trastuzumab to tumor progression.

10. Organ function levels must meet the following requirements:

5）Bone marrow function

- ANC ≥ 1.5×10^9/L；
- PLT ≥ 75×10^9/L；
- Hb ≥ 100 g/L；

6）Liver and renal function

- TBIL≤ULN；
- ALT and AST ≤ 3 × ULN (ALT and AST ≤ 5 × ULN for patients with liver metastases);
- BUN and Cr ≤ 1.5 × ULN and creatinine clearance ≥ 50 mL/min (Cockcroft-Gault formula);

7）Cardiac color ultrasonography

LVEF ≥ 50%；

8）12-lead ECG

QT interval ≤ 480 ms

11. Patients with known hormone receptor status.
12. Subjects who are willing to participate in this study sign the informed consent form, and have good compliance and willingness to cooperate with follow-up.

4.2 Exclusion criteria Subjects will be ineligible for enrollment into the study if any of the following criteria is met:

1. Patients with metastases to the central nervous system;
2. Inability to swallow, chronic diarrhea and intestinal obstruction, and multiple factors affecting drug intake and absorption;
3. Patients who have received radiotherapy, chemotherapy, surgical treatment (excluding local puncture) or molecular targeted therapy within 4 weeks prior to enrollment; those who have received anti-tumor endocrine therapy after screening.
4. Have participated in other drug clinical studies within 4 weeks prior to screening;
5. Prior or ongoing use of tyrosine kinase inhibitors targeting HER2

(lapatinib, lenatinib, pyrotinib, etc.)

6. Other malignancies within the past 5 years, excluding cured carcinoma in situ of the cervix, basal cell carcinoma of the skin, thyroid cancer, or squamous cell carcinoma of the skin.
7. Concurrently receive any other anti-tumor therapy.
8. Known history of allergy to the drug components in this protocol; history of immunodeficiency, including positive HIV test, HCV, active hepatitis B, or other acquired and congenital immunodeficiency diseases, or history of organ transplantation.
9. History of any cardiac disease, including: (1) arrhythmia requiring medical treatment or clinically significant; (2) myocardial infarction; (3) heart failure; (4) any other heart disease judged by the investigator to be inappropriate for participation in this study, etc.
10. Female patients during pregnancy and lactation, female patients of childbearing potential who have a positive pregnancy test at baseline, or female patients of childbearing potential who are unwilling to take effective contraceptive measures throughout the study.
11. At the discretion of the investigator, there are concomitant diseases that seriously endanger the safety of the patient or affect the completion of the study (including but not limited to serious hypertension uncontrolled by drugs, serious diabetes mellitus, active infection, etc.).
12. The toxicity of prior therapy did not recover to Grade 0-1 (except alopecia).
13. Previous confirmed history of neurological or mental disorders, including epilepsy or dementia.
14. Concomitant use of CYP3A4 inhibitors or inducers or medications that prolong the QT interval.
15. Any other circumstance that, in the opinion of the investigator, the patient is not suitable for participation in this study.

4.3 Withdrawal Criteria

4.3.1 Subject Withdrawal Criteria

Withdrawal from this clinical study:

1. The subject voluntarily withdraws ICF at any time;

The subject should discontinue the study treatment, but continue follow-up as required by the study, in case of the following conditions:

1. Radiographic disease progression or clinical progression;
2. Toxicity could not be tolerated when pyrotinib is reduced to 240 mg/d;
3. One interruption of pyrotinib > 14 days or accumulative interruption in a cycle > 14 days;
4. There are any clinical adverse events (AEs), laboratory abnormalities, or other medical conditions resulting in that the subject may no longer benefit from continued treatment;
5. The subject gets pregnant during the study;
6. Use of prohibited drugs specified in the protocol;
7. Other reason for inability to continue the study treatment in the opinion of the investigator.

4.3.2 Handling of Withdrawn Subjects

Every effort must be made to complete the End of Treatment/Withdrawal Visit in accordance with the protocol. Safety follow-up, efficacy follow-up (if required), and survival follow-up should be performed as specified in the follow-up period for subjects who discontinue the study treatment.

The investigator may recommend or provide a new or alternative treatment to the patient based on the actual situation.

4.4 Removal Criteria

1. Subjects who do not meet the inclusion criteria and meet the exclusion criteria;
2. Subjects with incomplete data that affect the judgment of efficacy and safety;
3. Failure to use drugs according to the dose, method and course of treatment specified in this protocol that affect the judgment of drug efficacy;
4. Use of prohibited medications specified in the protocol.

4.5 Termination Criteria

The study may be prematurely terminated or suspended for sufficient reason. If the study is prematurely terminated or suspended, the sponsor should submit written notice of the reasons for early termination or suspension to relevant departments. The Principal Investigator must immediately report to the EC and provide the appropriate justification.

Discontinuation criteria for this study include, but are not limited to, the following:

1. Unexpected, significant, or unacceptable risks to subjects are found;
2. Major errors in the protocol are found during the implementation of the study;
3. The study drug/study treatment is ineffective, or it is meaningless to continue the study;
4. Completion of the study is extremely difficult due to reasons such as severe delay in subject enrollment or frequent protocol deviations.

5 Study Drugs

5.1 Overview of Study Drugs

5.1.1 Names and Sources

Pyrotinib Maleate Tablets (Pyrotinib), is the innovative marketed drug manufactured by Jiangsu Hengrui Pharmaceuticals Co., Ltd.

Docetaxel Injection: the GCP-qualified clinician of the participating clinical study institution will select the approved marketed product in China based on the drug availability and efficacy consistency. The specific drug name and source will refer to the package insert and should be recorded in the subject diary and original medical records.

5.1.2 Drug Product Dosage Form and Strength

1) Dosage Form: pyrotinib tablets

Strength: 80 mg

Packaging: 14 tablets/bottle, 100 tablets/bottle (strength 80 mg)

2) Dosage Form: docetaxel injection

Strength: commercially available

5.1.3 Storage Conditions

Pyrotinib Maleate Tablets are sealed and stored in a dry place below 25 °C for not more than one month after opening; the valid period of Pyrotinib Maleate Tablets is 12 months.

Docetaxel should be stored at 2-25 °C, protected from light, where the valid period of docetaxel 20 mg is 24 months. The storage condition and shelf life of marketed drug should be in accordance with the package insert.

5.2 Method of Administration

The following drug regimen (pyrotinib + docetaxel) is recommended for the initial treatment of all subjects. The date of D1 of each cycle is calculated as 21 days after the date of docetaxel administration in the previous cycle, and if docetaxel administration ends, every 21 days is a cycle after the last docetaxel dose date. If the visit is postponed for docetaxel, pyrotinib needs to be continued; if docetaxel administration ends, the drug should be administered every 21 ± 3 days based on the calculated visit date after the date of the last docetaxel administration:

Pyrotinib: 400 mg once daily orally within 30 minutes of breakfast for 21 consecutive days in a cycle until disease progression or patient intolerance.

Docetaxel: 75 mg/m^2 administered on D1 of each cycle of 21 days, for 6 cycles, by intravenous infusion (allowable range of routine use is ± 10%), and docetaxel could only be discontinued in case of disease progression or intolerable toxicity before completion of 6 cycles. After the end of the 6 th cycle, the patient and doctor will carefully consider and decide whether to

discontinue docetaxel.

In addition, in order to alleviate adverse reactions, except for contraindications, it is recommended that all subjects should be pretreated according to medical practice before receiving docetaxel treatment, such as oral glucocorticoid dexamethasone before docetaxel, 16 mg daily (8 mg twice daily) for 3 days starting from 1 day prior to infusion; the specific pre-treatment regimen is determined by the investigator according to the clinical situation.

The above medications may be adjusted based on the adverse reactions of the subjects according to the protocol. Subjects will continue the treatment until disease progression, intolerable toxicity, withdrawal of informed consent, or discontinuation at the discretion of the investigator. The cycle date will be determined from the date of the first dose of the subject. In case of any dose interruption, omission, or underdose of the study drug during the study, the treatment will continue according to the cycle specified in the protocol without supplementation or modification of cycle. However, it should be recorded in detail in the original data: if there is a drug omission, the time and the reason for the missed dose should be recorded in detail; if the drug is underdosed due to various reasons such as adverse drug reactions, it should be recorded in the subject's diary and original medical records.

5.3 Management of Common Adverse Events

When AEs occur during the study, the investigator should actively treat them symptomatically, and record the concomitant therapies and medications in detail in the disease course and in the EDC system.

Based on data from previous clinical studies of pyrotinib, pyrotinib monotherapy is well tolerated. The incidence of grade 3/4 AEs was 25% (2/8) in the 400 mg group and 11.1% (1/9) in the 320 mg group. Therefore, according to the type and time of clinical AEs in the study, the relevant drugs can be first adjusted or discontinued. The pyrotinib dose modification scheme is shown in Table 4 and Table 5; docetaxel monotherapy or pyrotinib monotherapy is allowed. The investigator should perform medical treatment based on the clinical situation, and the following treatment methods are provided for reference:

1) Allergic reactions: mild rash, skin flushing or pruritus after the treatment should be observed without special treatment; if moderate rash and flushing, mild dyspnea, or chest discomfort occur, the drug needs to be discontinued, and symptomatic anti-allergic treatment should be given as appropriate according to the doctor's assessment, such as injection of 10 mg dexamethasone or 40 mg methylprednisolone, and the infusion rate should be slowed after the symptoms are relieved. If there are recurrent allergic symptoms during the re-administration, it is recommended to discontinue the drug. If severe symptoms such as hypotension requiring blood pressure raising therapy, angioneurotic edema, respiratory distress requiring

bronchodilator therapy, and generalized urticaria, the drug should be discontinued immediately and active symptomatic treatment should be given, with additional fluid replacement, vasopressin and bronchodilator as appropriate. After stabilization of symptoms, the drug can be continued under close observation after careful evaluation by the doctor. The infusion rate can be controlled at 10 mL/hour and increased to 25 mL/hour after 15 minutes. If the subject has no discomfort after 15 minutes, the infusion can continue to be completed at the usual rate.

2) Diarrhea: before the subject starts oral administration of the study drug, the investigator should inform in detail the possibility of diarrhea and the management measures for diarrhea. Symptomatic treatment and close follow-up or observation (≤ 14 days) should be performed in the event of diarrhea. In the event of Grade 1-2 diarrhea, diet should be adjusted, appropriate drug intervention (e.g., probiotics, montmorillonite powder, loperamide, etc.) should be given, and dose reduction in the treatment regimen is not required; for Grade 3 diarrhea or Grade 1-2 diarrhea with complications that remain unresolved, docetaxel is recommended to be interrupted first; if the diarrhea is still not resolved after docetaxel interruption, pyrotinib should be interrupted at the discretion of the investigator. The dosing may be resumed as specified in Table 3 and Table 4 after the AE is recovered to Grade 1.

3) Hand and foot skin reaction and rash: symptomatic treatment should be given first, and close follow-up should be performed. Suggested symptomatic supportive care: strengthen skin care, and keep skin clean to avoid secondary infections; avoid pressure or friction; use emollients or lubricants, and topically use emulsions or lubricants containing urea and corticosteroids; and topically use antifungal or antibiotic therapy as appropriate. For Grade 2 hand-foot syndrome that remains unresolved, docetaxel interruption is recommended first; if the hand-foot syndrome is still not resolved after docetaxel interruption, pyrotinib should be interrupted at the discretion of the investigator. The dosing may be resumed as specified in Table 4 and Table 5 after the AE is recovered to Grade 1.

4) Hepatic function abnormal: symptomatic hepatoprotective treatment or observation should be given by the investigator based on the subject's condition and AE, and the frequency of blood chemistry tests should be increased as clinically indicated. If the hepatic function abnormal ≥ Grade 2 is still present after active treatment or observation (≤ 14 days), the drugs should be interrupted (docetaxel is recommended to be interrupted first at the discretion of the investigator). The original dose will be resumed after the AE is recovered to Grade 1. At the discretion of the investigator, dose reduction may also be performed as appropriate. For subjects with liver metastases, if ALT/AST > 3 × ULN at enrollment, liver function should be closely monitored to determine whether the subject is suitable for enrollment in the study based on comprehensive considerations. In addition, if ALT and/or AST > 1.5 × ULN with alkaline

phosphatase > 2.5 × ULN, the recommended dose of docetaxel is 75 mg/m^2; if serum bilirubin > the upper limit of normal and/or ALT and AST > 3.5 × ULN with alkaline phosphatase > 6.0 × ULN, docetaxel should not be used and there is no recommendation for dose reduction, unless strictly indicated for use.

5) Neutropenia/thrombocytopenia: hematology should be tested before the subject starts the study drug treatment. If the neutrophil count is less than 1500/mm^3 and the platelet count is less than 75 × 10^9/L, docetaxel cannot be used. If febrile neutropenia occurs and the neutrophil count < 500/mm^3 persists for more than 1 week during treatment, it is recommended to first reduce or discontinue docetaxel, and prophylactic use of G-CSF (e.g., Day 4 to 11) is recommended in subsequent cycles. If subjects continue to experience the above-mentioned reactions, G-CSF should be continued, and the docetaxel dose should be reduced to 60 mg/m^2. For neutropenia and/or thrombocytopenia that remains unresolved after docetaxel interruption, pyrotinib should be interrupted at the discretion of the investigator. The dosing may be resumed as specified in Table 3 and Table 4 after the AE is recovered to Grade 1.

6) Vomiting: symptomatic treatment should be given first, and close follow-up should be performed. For Grade 2 or higher vomiting that is still not relieved, the drugs should be interrupted at the discretion of the investigator (docetaxel is recommended to be interrupted first at the discretion of the investigator); the original dose will be resumed after the AE recovers to Grade 1. If the time of vomiting is close to the time of dosing on the same day, the onset time of vomiting should be recorded in detail. However, regardless of whether vomiting affects the absorption of the study drug, the drug will continue to be taken according to the protocol thereafter, without supplementation or cycle modification.

7) Cardiotoxicity: asymptomatic sinus bradycardia or tachycardia generally does not require special treatment. If ventricular tachycardia and coupled heart beats occur, the drugs should be discontinued immediately and the subject should be managed according to standard arrhythmic treatment regimens, such as lidocaine 1-1.5 mg/kg (generally 50-100 mg) intravenous infusion as the loading dose, followed by intravenous infusion at a rate of 1-4 mg/min or amiodarone 150 mg IV, followed by 900 mg maintenance for 24 hours. Severe AV block may be treated with atropine 1-2 mg or isoproterenol 0.5-1.0 mg IV, and a temporary cardiac pacemaker should be installed immediately if necessary, which should be handled by the study doctor in accordance with clinical practice and experience. Refer to Table 5 for treatment regimens of conditions leading to LVEF decline.

8) Neurotoxicity: general peripheral neurotoxicity does not need to discontinue the drugs, but the dosage should be reduced according to Table 4 in subsequent treatments. It can be improved spontaneously within a few months after drug withdrawal. Paresthesias is usually prevented

and ameliorated with neurotrophic drugs, such as amifostine, glutamine, acetyl-L-carnitine, and vitamin E. Treatments of neuropathic pain include various tricyclic antidepressants, anticonvulsants, serotonin reuptake inhibitors, and nonsteroidal anti-inflammatory drugs. In the event of grand mal seizures, the drugs should be discontinued immediately, and diluted diazepam 10 mg should be given by slow intravenous bolus. After all neurotoxicity occurs, it is advised to consult the neurologists to assist in assessing the condition and actively managing symptoms. The dosing may be resumed as specified in Table 4 and Table 5 after the AE is recovered to Grade 1.

5.4 Dose Modification Scheme

5.4.1 Dose Interruption and Reduction of Docetaxel

AEs should be closely monitored during treatment, and adverse reactions caused by docetaxel can be managed by symptomatic treatment, drug discontinuation, and dose modification. For AEs that cannot be controlled after treatment or observation (≤ 14 days) in the study, the investigator may preferably suspend docetaxel based on the type and timing of the clinical AEs. For the dose interruption and reduction of docetaxel, please refer to the drug package insert. Once the dose of docetaxel is reduced, it cannot be increased later or the decision to increase the dose will be made after assessment by the investigator. For specific dose modification scheme, please refer to Table 4.

Table 4 Dose Interruption and Modification Scheme of Docetaxel

NCI-CTC AE 5.0	Handling of Treatment Process	Dose Modification for Next Cycle
Grade 1	Maintain the original dose	Maintain the original dose
Grade 2		
1st occurrence	Interruption until recovery to Grade 0-1	Docetaxel 75 mg/m^2
2nd occurrence	Interruption until recovery to Grade 0-1	Docetaxel 60 mg/m^2
3rd occurrence	Interruption until recovery to Grade 0-1	Docetaxel 50 mg/m^2
4th occurrence	Permanent discontinuation	
Grade 3		
1st occurrence	Interruption until recovery to Grade 0-1	Docetaxel 75 mg/m^2
2nd occurrence	Interruption until recovery to Grade 0-1	Docetaxel 60 mg/m^2
3rd occurrence	Permanent discontinuation	
Grade 4	Permanent discontinuation<br>Or if, in the opinion of the investigator, continuation of treatment is in the best interest of the patient, the dose should be interrupted until AE resolution to Grade 0 - 1, then the patient may continue the treatment.	

* Grades of hand and foot skin reaction and other AEs not included are provided in 9.5.2. The investigator should give clinically active treatment or observation (≤ 14 days) based on the subject's condition and AE,

and if the AE is still present, it is recommended to adjust the drug according to this table.

5.4.2 Dose Interruption and Reduction of Pyrotinib

The dose of pyrotinib may be interrupted and adjusted by the investigator according to the type, severity and time of AEs, and may be discontinued in case of SAEs. In the study, the dose of pyrotinib is allowed to be interrupted and adjusted for multiple times, and the dose of pyrotinib will be adjusted in the gradient of 400 mg, 320 mg, and 240 mg.

After dose modification, if the subject still has Grade ≥ 3 diarrhea or Grade 1-2 diarrhea with complications or other ≥ Grade 2 AEs that are clinically uncontrollable (i.e., persisting after ≤ 14 days of clinical treatment or observation, with ≥ 2 occurrences), another dose reduction of a gradient is allowed at the resumption after interruption, with minimum dose of pyrotinib 240 mg, as judged by the investigator.

Multiple drug interruptions are allowed during the treatment, and each interruption should be revoked after the AE recovers to Grade 0-1 and the complications disappear. Cumulative interruption of pyrotinib should not exceed 14 days per cycle and each interruption of pyrotinib should not exceed 14 days to ensure the drug intensity of the subject treatment. If the interruption of pyrotinib due to an AE exceeds the above time limit, the subject will be discharged from the study.

Table 5 Pyrotinib Dose Modification Requirements

NCI-CTC AE 5.0	Handling of Treatment Process (After active clinical treatment or observation)	Dose Modifications for Resumption
Cardiac Toxicity		
Clinically significant ≥ Grade 2 decrease in LVEF/LVEF below the lower limit of normal (including asymptomatic decrease in LVEF ≥ 10% and LVEF < 50%, or heart failure)	Permanent discontinuation	-
Diarrhea		
Grade 4	Permanent discontinuation	-
Grade 3	Interruption until recovery to Grade 0-1 and resolution of complications	First time 400 mg<br>Second time 320 mg<br>Third time 240 mg
Grade 1 to 2 with complications (including but not limited to mild to severe abdominal colic, ≥ Grade 2 nausea or vomiting, decreased ECOG score, fever, sepsis, neutropenia, hemorrhage, or dehydration)		
Other AEs		
≥ Grade 2 nonhematological AEs (except alopecia, fatigue, asthenia, etc.)	Interruption until recovery to Grade 0-1	First time 400 mg<br>Second time 320 mg<br>Third time 240 mg
≥ Grade 3 hematological AEs	Interruption until recovery to	First time 400 mg

	Grade 0-1	Second time 320 mg<br>Third time 240 mg

LVEF, Left Ventricular Ejection Fraction.

* Grades of hand and foot skin reaction and other AEs not included are provided in 9.5.2. The investigator should give clinically active treatment or observation (≤ 14 days) based on the subject's condition and AE, and if the AE is still present, it is recommended to adjust the drug according to this table.

5.5 Management, Dispensing and Return of Study Drugs

The management, dispensing and return of study drugs in this study are the responsibility of a special person. The investigator must ensure that all study drugs are used only in the subjects participating in the clinical study, the dosage and usage will comply with the protocol, the unused drugs will be returned to the study site, and the study drugs will not be transferred to any non-clinical study participants.

The study drugs will be stored at room temperature. A drug receipt form must be signed at the time of drug dispensing. At the end of the study, the unused drugs and empty boxes will be recovered and the drug return form will be signed. Dispensing and return of each study drug should be timely documented in the corresponding record sheet.

The CRA is responsible for monitoring the supply, use, and storage of the study drugs and disposal of unused study drugs.

5.6 Compliance with Study Drug Administration

After the study drug is dispensed to an individual subject, the subject should be asked to return unused study drug at each subsequent visit. The subject should record medication information in a diary every day.

Compliance with the study drug administration (e.g., taking the specified dose every day) will be evaluated at each clinical visit (except the follow-up visit) while the subject is taking the drug. At each study visit, the site personnel will compare the returned study drug and subject-reported dose information and compare to the prescribed dose in order to monitor compliance. The number of returned study drugs will be recorded. Deviations between the number of returned study drug and subject-reported dosing information should be reconciled with the subject during the visit. Compliance and unidentified deviations will be recorded in the source documents as well as in the drug inventory records. Any deviations from compliance should be documented and explained accordingly.

Calculation of dose compliance:

% Compliance = Number of tablets taken/number of tablets expected to be taken × 100.

A subject may be considered noncompliant with quantitative dosing if the percent compliance calculated by the above formula is less than 80% or greater than 120%. If a subject’s compliance is outside the above range, it will be recorded as a protocol deviation. The study drug eCRF

should reflect the verified dose information provided by the subject.

6 Concomitant Medications

6.1 Prohibited Medications During the Study

Anti-tumor drugs and adjuvant drugs related to tumor therapy should be discontinued during treatment, including traditional Chinese medicines and traditional Chinese medicinal products, immunological agents, etc.

6.2 Medications to be Used with Caution During the Study

In case of adverse reactions, the subjects should be closely observed, active symptomatic treatment should be given if necessary, and the medications used should be recorded and explained on the CRF form. The following medications will be used with caution during the study:

- Drugs interfering with hepatic cytochrome P450 enzymes:

1. CYP3A4 inducers (dexamethasone, phenytoin sodium, carbamazepine, rifampin, rifabutin, rifapentine) and inhibitors (ketoconazole, itraconazole, erythromycin, cyclosporine, clarithromycin, etc.);
2. Substrates of CYP3A4 (simvastatin, pimozide);
3. Other drugs metabolized by CYP3A4 (e.g., benzodiazepines, dihydropyridine, calcium antagonists, and HMG-COA reductase inhibitors);
4. Substrates of CYP2C9 (diclofenac, phenytoin sodium, piroxicam, S-warfarin, and tolbutamide) and CYP2C19 (diazepam, imipramine, lansoprazole, and S-mephenytoin).

- Food interfering with liver P450 enzymes:
  Grapefruit, black mulberries, wild grapes, pomegranates, black raspberries, and beverages and food containing the above fruit components.
- Drugs that prolong the QT interval: include antibiotics, antiarrhythmics, antipsychotics, antifungals, antimalarials, and antidepressants (e.g., clarithromycin, quinidine, risperidone, fluconazole, mefloquine, amitriptyline, azithromycin, sotalol, fluphenazine, ketoconazole, chloroquine, imipramine, erythromycin, amiodarone, droperidol, clomipramine, roxithromycin, disopyramide, haloperidol, dothiepin, metronidazole, procainamide, thioridazine, doxepin, moxifloxacin, pimozide, olanzapine, clozapine, etc.)

6.3 Allowed Concomitant Medications and Treatments During the Study

Patients may receive supportive care, and clinical comorbidities and AEs should be actively treated. All concomitant medications should be recorded in the eCRF in strict accordance with

GCP regulations. Concomitant medications/treatments should be recorded from 4 weeks prior to study treatment until AE resolution, other anti-tumor therapy, or death, whichever occurs first.

6.4 Prophylactic Antidiarrheal

Because of the different incidence of diarrhoea with TKI drugs, the prevention of diarrhea also varies. Lapatinib has a low incidence of diarrhea and antidiarrheal prophylaxis is not mandatory. Primary prevention of diarrhea is recommended for pyrotinib, antidiarrheal prophylaxis is recommended during the first 2 cycles (42 days) of treatment, and loperamide prophylaxis should be initiated at the first dose of pyrotinib. Patients are instructed to take loperamide as shown in Table 6 and to adjust the frequency of use to control bowel movements to 1-2 times daily.

Table 6: Loperamide Prophylaxis

Time of Pyrotinib Administration	Loperamide Dose Frequency
Week 1-2 (Day 1-14)	2 mg, three times daily
Week 3 (Day 15-21)	2 mg, twice daily
Week 4-6 (Day 22-42)	2 mg, twice daily
Week 7-Disease progression (Day 43-PD)	2 mg, as needed, not more than 16 mg/day

Diarrhea can be controlled by temporarily interrupting pyrotinib or reducing its dose as clinically indicated, with dose modifications shown in Table 6.

6.5 Prophylactic Leukocyte Elevation

As the risk of neutropenia is significantly associated with chemotherapy, this study recommends prophylactic leukocyte elevation for the pyrotinib combined with docetaxel regimen and it should be initiated at the first dose of docetaxel until docetaxel is discontinued for any reason.

Recommended drugs for leukocyte elevation: 艾多®, Mecapegfilgrastim Injection.

Dosage: fixed dose of 6 mg each time, or individualized treatment at 100 μg/kg based on patient weight.

Method of administration: subcutaneous injection.

Dosing time: 24-48 h after the end of chemotherapy, one injection per chemotherapy cycle.

If a patient develops ANC < 0.5 x 10^9/L and/or febrile neutropenia lasting 2 days after treatment with mecapegfilgrastim, G-CSF 5 ug/kg should be administered subcutaneously every day until peripheral blood ANC is > 5.0 x 10^9/L. Antibiotic prophylaxis is not indicated. Antibiotics may be used in the event of FN, definite infection, or temperature of 38 °C and infectious fever cannot be ruled out. If a patient develops ANC < 0.5 x 10^9/L and/or FN lasting 2 days, G-CSF 5 ug/kg should be administered subcutaneously every day until peripheral blood ANC is > 5.0 x 10^9/L.

Dose modification or delay

If a patient experiences a serious adverse reaction, the dose needs to be adjusted or delayed in the next cycle.

If the toxicity recovers to Grade 1/2 according to CTCAE 5.0, the chemotherapy will be continued; otherwise, the chemotherapy will be delayed. For toxic reactions (e.g., taste changes, etc.) that, in the opinion of the investigator, are unlikely to cause a serious or life-threatening event, the study medication should not be reduced or interrupted and should be continued at the original dose. In addition, toxic reactions that can be satisfactorily controlled by symptomatic management or prophylaxis do not require to reduce, delay, or discontinue the treatment.

In the event of serious hematological and non-hematological toxicities, the drug dose causally related to the toxicity should be adjusted. If multiple toxicities occur in a treatment cycle, the parameter for this dose modification will be based on the highest grade of toxicity.

7 Study Procedures

Subjects must read and sign the ICF currently approved by the EC before starting the study. All study procedures will be performed within the time window specified in the study schedule.

7.1 Screening Period

After signing the informed consent form, subjects will enter the screening period. Unless specifically noted, the following screening procedures must be completed within 28 days prior to the study period:

- Demographic data collection: initials, sex, ethnicity, marital status, date of birth, height, weight, and body surface area will be calculated accordingly;
- Medical history inquiry: past medical history and treatment history (clinical/pathological diagnosis, time of diagnosis, clinical/pathological stage, HER2/ER/PR/expression; whether the patients have received surgery, neoadjuvant therapy, adjuvant therapy, or radiotherapy; progress time and rationale for diagnosis of progression; drug name, dosage and administration, start and end time of treatment, cycle, and efficacy of each line of advanced systemic therapy; whether applying other treatment, such as surgery), smoking and alcohol consumption history (frequency, amount, duration), drug allergy history (drug name, allergic symptoms), prior or concomitant diseases/symptoms (disease/symptom name, concomitant medication name, dose, usage, outcome), and stool habits (number of times);
- ECOG score;
- Vital signs: body temperature, blood pressure, respiratory rate, pulse;
- Physical examination: general condition, skin and mucosa, lymph nodes, head and neck,

chest, abdomen, muscles and bones;

- Neuroreflexes, respiratory system, cardiovascular system, genitourinary system, mental status, etc.;
- Hematology;
- Urinalysis;
- Stool routine;
- Blood chemistry;
- Coagulation function: including prothrombin time (PT), activated partial thromboplastin time (ATTP), thrombin time (TT), and national normalized ratio (INR); once at the screening period;
- Infectious disease screening;
- Pregnancy test;
- 12-lead ECG: 3 examinations (at least 10 minutes apart is recommended) at screening, and the mean of the 3 QTcFs will be used as the baseline value;
- Cardiac color ultrasonography: all reports within 28 days prior to study drug administration may be used (including qualified cardiac color ultrasonography completed before signing the ICF);
- Tumor imaging: all reports within 28 days prior to study drug administration may be used (including qualified tumor imaging completed before signing the ICF);
- Concomitant medications/treatments: concomitant medications during the screening period and during the study should be recorded; once the subject interrupts the study treatment, only concomitant medications and therapies for new or unresolved AEs related to the study treatment should be recorded;
- Follow-up of AEs: AEs will be recorded from the day the subject signs the informed consent form until 28 days after the last dose; after completing all the above screening assessments, eligible subjects will obtain the enrollment number.

7.2 Study Period

After obtaining the enrollment number, the examinations will be completed according to the time windows listed in the study procedures. In case of a legal holiday, the examinations can be advanced accordingly and the reasons for the out-of-window should be recorded in the eCRF. The date of D1 of each cycle is calculated as 21 days after the date of docetaxel administration in the previous cycle. If the dose of docetaxel is delayed as specified in the protocol, the postponement of the visit date is not a protocol deviation; if the docetaxel administration ends, the visit date thereafter is calculated according to the date of the last docetaxel dose. The visit date is fixed and does not change according to the actual visit date. Visit at the end of Week 1,

2 and 3 of Cycle 1 (D7 ± 3, D14 ± 3, D21 ± 3) and at the end of Week 1, 2 and 3 of Cycle 2 (D7 ± 3, D14 ± 3, D21 ± 3); thereafter once at the end of each cycle (D21 ± 3) until Cycle 12; once at the end of every 2 cycles (D21 ± 3) for Cycle 13 to 36; and once at the end of every 4 cycles (D21 ± 3) after Cycle 37.

The investigator may add tests or increase the frequency of visits based on the subject's clinical condition:

- ECOG score: assessed at the end of every 2 cycles within 36 cycles and every 4 cycles after Cycle 37;
- Vital signs: once at the end of Week 1, 2 and 3 of Cycle 1 (D7 ± 3, D14 ± 3, D21 ± 3), once at Week 1, 2 and 3 of Cycle 2 (D7 ± 3, D14 ± 3, D21 ± 3), thereafter once every cycle until Cycle 12 (D21 ± 3), every 2 cycles in Cycle 13 to 36 (D42 ± 3), and every 4 cycles after Cycle 37 (D84 ± 3);
- Physical examination: at the end of every 2 cycles within 36 cycles and every 4 cycles after Cycle 37;
- Hematology: once at Week 1, 2 and 3 of Cycle 1 (D7 ± 3, D14 ± 3, D21 ± 3), once at Week 1, 2 and 3 of Cycle 2 (D7 ± 3, D14 ± 3, D21 ± 3), thereafter once every cycle until Cycle 12 (D21 ± 3), every 2 cycles in Cycle 13 to 36 (D42 ± 3), every 4 cycles after Cycle 37 (D84 ± 3), and test in another hospital may be acceptable;
- Urinalysis: if urinalysis shows urine protein ++ or above, please add a 24-h urine protein quantification test at the discretion of the investigator;
- Stool routine: tested once at the end of each cycle (test in another hospital may be acceptable);
- Blood chemistry: once at Week 1, 2 and 3 of Cycle 1 (D7 ± 3, D14 ± 3, D21 ± 3), once at Week 1, 2 and 3 of Cycle 2 (D7 ± 3, D14 ± 3, D21 ± 3), thereafter once every cycle until Cycle 12 (D21 ± 3), every 2 cycles in Cycle 13 to 36 (D42 ± 3), and every 4 cycles after Cycle 37 (D84 ± 3); myocardial zymogram may be performed if necessary, at the discretion of the investigator according to the subject's condition;
- 12-lead ECG: once at Week 1, 2 and 3 of Cycle 1 (D7 ± 3, D14 ± 3, D21 ± 3), once at Week 1, 2 and 3 of Cycle 2 (D7 ± 3, D14 ± 3, D21 ± 3), thereafter once every cycle until Cycle 12 (D21 ± 3), every 2 cycles in Cycle 13 to 36 (D42 ± 3), and every 4 cycles after Cycle 37 (D84 ± 3). During the study, if the QTc interval increases >30 msec from baseline, or the absolute value of QTc interval ≥ 480 msec occurs in any specified ECG measurement, two additional ECGs (at least 10 minutes apart is recommended) are required (examination in another hospital is acceptable).
- Cardiac color ultrasonography: if symptoms such as chest pain and palpitations occur

during the study, additional examinations may be performed as appropriate; it will be performed at the end of every 2 cycles after enrollment;

- Tumor imaging: tumor imaging time points during the dosing period will be determined after the start of study treatment, regardless of the time of dose interruption due to toxicity during this period. The allowed time window for tumor imaging is ± 7 days, and the specific assessment time points are as follows:
  - ✓ Every 6 weeks (regardless of treatment delay);
  - ✓ In the event of radiologically confirmed PD, the subject will discontinue the study treatment and enter the follow-up period. No other anti-tumor therapy can be performed prior to PD;
- Concomitant medications/treatments: concomitant medications/treatments during the study will be recorded at any time;
- AEs: AEs during the study will be observed and recorded at any time.
- Drug return/dispensing: drug return and dispensing will be recorded.

7.3 End of Treatment/Withdrawal from the Study

Subjects will continue the treatment until disease progression, intolerable toxicity, withdrawal of informed consent, or discontinuation at the discretion of the investigator. At the end of treatment or withdrawal from the study, If a patient does not have the following examinations or tests within 7 days prior to the end of study (except for tumor imaging assessments), the following examinations or tests should be performed within 7 days after withdrawal from the study.

- ECOG score;
- Vital signs;
- Physical examination;
- Hematology: if not performed within 7 days;
- Urinalysis: if not performed within 7 days;
- Stool routine: if not performed within 7 days;
- Blood chemistry: if not performed within 7 days;
- Pregnancy test;
- 12-lead ECG: if not performed within 7 days;
- Cardiac color ultrasonography: if not performed within 4 weeks;
- Tumor imaging: if not performed within 4 weeks;
- Concomitant medications/treatments: real-time recording;
- AEs: real-time recording.
- Drug return: recover any unused drug in the study.

7.4 Follow-up Period

Subjects will enter the follow-up period from the day after the last dose of study drug, and the following follow-up visits will be performed until the survival follow-up is completed (no follow-up is required for subjects enrolled without treatment):

- Safety follow-up: SAEs will be followed up to 30 days after the last dose of study drug, and AEs will be followed until complete resolution or being considered by the investigator to be non-responsive and stable, or initiation of other anti-tumor therapy, whichever occurs first.
- Efficacy follow-up: subjects who withdraw from the study due to non-PD and non-death causes should be followed for efficacy. Tumor imaging assessment continues at the time points specified in the protocol (at the end of every 2 cycles within 20 cycles and at the end of every 4 cycles after 21 cycles) from the last tumor imaging assessment during the study until PD, initiation of other anti-tumor drugs, or death, whichever comes first. Each follow-up time, tumor imaging assessment results and other anti-tumor treatment information during this period will be recorded in detail.
- Survival follow-up (OS data collection): all survived subjects who complete the safety follow-up and efficacy follow-up (whichever is later) need to undergo survival follow-up. Survival information (date and cause of death) and information after the study treatment (including the received treatment) will be collected by telephone or clinical follow-up of the subject, his/her family or local doctor at least once every 12 weeks (± 7 days) from the date of completion of the safety follow-up visit and efficacy follow-up visit (whichever is later) until death, subject is lost to follow-up, or the end of OS data collection, whichever occurs first.

8 Efficacy Evaluation

8.1 Imaging

High-resolution contrast-enhanced CT or contrast-enhanced MRI is recommended for tumor imaging. Subjects with a history of contrast allergy should be treated according to the guidelines for the prevention of contrast allergy at the study site to perform contrast-enhanced CT or contrast-enhanced MRI whenever possible. If contrast is contraindicated for a subject, plain CT and MRI scans are allowed.

At least chest, abdomen, and craniocerebral examinations will be included at screening, while neck and pelvic examination, bone scan, etc. may be added as clinically indicated. The investigator may add scan sites in tumor assessments at or after baseline as clinically indicated. Imaging results such as CT/MRI scans obtained before signing the informed consent can be

used for screening assessments as long as they meet the requirements (within 28 days prior to the first dose of study drug).

Subsequent imaging assessments should be performed under the same conditions as the baseline examination (slice thickness of the scan, use of contrast, etc.). Tumor imaging time points during the dosing period will be determined after the start of study treatment, and dose interruption will not change the evaluation time. The allowed time window for tumor imaging is ± 7 days, and the specific assessment time points are as follows:

- Every 6 weeks (regardless of treatment delay); for patients with bone metastases at baseline, bone scans will be performed at least once every 6 months (approximately 180 days) after the start of dosing;

Bone scans and PET are not appropriate for response assessment of target lesion per RECIST 1.1 criteria. If necessary, these examinations can be used to assess non-target lesions, but the frequency of assessments of these non-target lesions may be reduced. For example, bone scans may be repeated only in case of confirmed CR in target lesions or suspected progression in bone lesion.

Imaging assessments for this study will be performed at each site (on-site review) by an experienced qualified study doctor designated by each site. In addition, all imaging data related to efficacy evaluation should be archived on CD-ROM at each study site. Imaging assessments will be performed at each site and central imaging assessments will be performed by the central laboratory. Response evaluation based on tumor imaging will be performed by the study site according to RECIST 1.1 criteria.

8.2 Primary Endpoint Measure

Objective response rate (ORR): the percentage of subjects with best response of CR or PR in the overall analysis dataset from the start of the study treatment until the subject's disease progression and withdrawal from the study. Objective tumor response is assessed using RECIST 1.1 criteria. Subjects must have measurable tumor lesions at baseline. Response assessment criteria are categorized as complete response (CR), partial response (PR), stable disease (SD, stable disease ≥ 24 weeks), and disease progression (PD) according to RECIST 1.1 criteria.

8.3 Secondary Endpoint Measures

Overall survival (OS) is defined as the time from signing ICF to death due to any cause. For subjects who are still alive at the last follow-up, their OS will be considered to be censored at the last follow-up time. For subjects who are lost to follow-up, their OS will be considered to be censored at the last follow-up time they are known to be alive before lost to follow-up. Censored OS is defined as the time from enrollment to censoring.

Progression-free survival (PFS) is defined as the time from signing ICF until the first radiographic assessment of disease progression (PD) or death due to any cause. If the subject does not experience PD or death at the cut-off date, or have received other anti-tumor therapy, the last response assessment before the cut-off date or the start date of other anti-tumor therapy, whichever occurs first, will be used as the censoring time.

Clinical benefit rate (CBR) is defined as the percentage of patients with confirmed complete response, partial response, and stable disease (≥ 24 weeks) in efficacy evaluable patients.

Duration of response: the overall duration of response is defined as the time from the first assessment of CR/PR, whichever occurs first, to relapse or PD.

All the above measures will be assessed according to RECIST 1.1 criteria except OS, and the analysis of the measures will include tumor evaluation results during the study treatment and follow-up period. If a patient has several measures that can be judged to be PD, the first measure will be used for the analysis of ORR, DoR, etc. Recurrence, new lesions, or death are considered to reach the study endpoint, and the use of other systemic or targeted anti-tumor therapies for the target lesions is also considered as tumor progression.

9 Safety Evaluation

During the study, the safety of the compound will be evaluated by AE records (including SAEs), laboratory tests, vital signs, physical examination, ECOG score, cardiac color ultrasonography, and ECG records. During the study, subjects should be closely observed for symptoms and signs after dose. Adverse events/reactions should be managed timely and effectively to ensure the safety and interests of subjects. After timely and effective management of adverse events/reactions, the type, symptoms, time of onset, degree (or grade), symptomatic treatment methods and outcome should be recorded, and then AEs should be analyzed, assessed and statistically analyzed as the basis for the continuation of the study.

9.1 Physical Examination and Vital Signs

The physical examination will be performed by the study doctor and include: general condition, skin and mucosa, lymph nodes, head and neck, chest, abdomen, muscles and bones, neuroreflexes, respiratory system, cardiovascular system, genitourinary system, mental status, etc. Weight will be measured at each physical examination, but height only needs to be measured once at screening.

Vital signs include the following: body temperature, blood pressure, respiratory rate, and pulse.

The ECOG score will be rated by the study doctor according to Appendix I, "Performance Status Scoring Criteria (ECOG)".

9.2 Laboratory Tests

Laboratory samples will be collected according to the time points specified in the "Clinical Study Flow Chart". As shown in Table 7, the following laboratory parameters will be sampled and tested by the study site. Unscheduled clinical laboratory tests may be performed at any time for the safety of subjects.

Table 7 Requirements for Laboratory Tests

Hematology	Blood chemistry	Stool routine	Urinalysis [a]
Hemoglobin<br>Red blood cells<br>White blood cells<br>Neutrophil count<br>Lymphocyte count<br>Platelet count	Total bilirubin<br>Direct bilirubin<br>Indirect bilirubin<br>ALT<br>AST<br>Alkaline phosphatase<br>r-GT<br>Total protein<br>Albumin<br>Urea nitrogen<br>Creatinine<br>Uric acid<br>Blood glucose<br>Triglycerides<br>Cholesterol<br>Potassium<br>Sodium<br>Cl<br>Calcium<br>Magnesium<br>Phosphorus	Stool appearance<br>Fecal occult blood	Urine protein<br>Urine glucose<br>Urinary occult blood
Infectious disease screening	Others		
Hepatitis B five items<br>HIV antibody<br>HCV antibody	Pregnancy test [b]		

Notes: a. If the semiquantitative method shows protein ≥ 2+ (e.g., dipstick), a 24-hour urine protein quantification test should be performed.

b. Women of childbearing potential should undergo blood HCG testing at screening to exclude pregnancy, and urine HCG testing may be performed at other time points.

9.3 Electrocardiogram

The 12-lead ECG will be performed by a qualified doctor according to the time points specified in the clinical study flow chart. All ECGs are required to be performed after the subject has rested quietly in a supine position for at least 10 minutes. ECG should include at least: heart rate, QT, QTcF and P-R interval. Three examinations (at least 10 minutes apart) will be performed at screening, and the mean of the 3 QTcFs will be used as the baseline value.

To assess the safety of subjects, the study doctor will compare ECG results with baseline measurements. If the QTc interval increases by > 30 msec from baseline, or if any of the specified ECG measurements shows an absolute QTc interval ≥ 480 msec, two additional ECGs are required at least 10 minutes apart to determine the accuracy of the original measurement

and exclude abnormal ECGs due to incorrect lead placement. If a machine-read QTc value is prolonged as defined above, repeat measurements may not be performed provided that a qualified physician determines that the QTc value is in the acceptable range.

9.4 Cardiac Color Ultrasonography

Cardiac color ultrasonography will be performed by a qualified doctor according to the time points specified in the clinical study flow chart. During administration of the study drug, LVEF will be assessed and monitored by the study doctor at the time points specified in the protocol. During the administration, subjects with symptoms of heart failure or clinically significant decrease in LVEF should be treated and monitored according to standard medical guidelines at the discretion of the investigator, and consult a cardiologist if necessary. In the event of clinically uncontrolled symptoms of severe heart failure (NYHA Class III or IV) or significant decrease in LVEF (below the lower limit of normal or less than 50%), the study drug should be discontinued as specified in Section 5.4 Dose Modification Scheme and the subject should continue to be treated and monitored in accordance with standard medical guidelines.

9.5 Adverse Events (AEs)

9.5.1 Definition of Adverse Events

An AE is any untoward medical occurrence in a clinical study after the subject signs the informed consent form, which does not necessarily have a causal relationship with the study drug. An AE may be any unexpected, unfavorable symptom, sign, disease, or abnormal test result, whether it is related to the study drug or not. AEs include the following: 1) medical conditions/diseases that are present before the start of study treatment and worsen after the start of study drug; 2) any new AEs; 3) abnormal changes in laboratory test results that are considered clinically significant.

9.5.2 Criteria for Determining the Severity of Adverse Events

Refer to NCI-CTC AE Version 5.0 for the grading criteria for AEs.

For AEs not listed in the NCI-CTC AE Version 5.0, the following criteria can be used:

Grade I: Mild; asymptomatic or minimal clinical symptoms; clinical or diagnostic observations only; no treatment indicated;

Grade II: Moderate; minimal, local or noninvasive intervention indicated; limiting age-appropriate instrumental activities of daily living (ADL). Instrumental ADL refers to preparing meals, shopping, using the telephone, managing money, etc.

Grade III: Severe or medically significant but not immediately life-threatening; requiring hospitalization or prolonged hospitalization; resulting in disability; resulting in limiting self-care ADL. Self-care ADL refer to bathing, dressing, undressing, feeding self, using the toilet, taking medications, etc., and not bedridden.

Grade IV: Life-threatening consequences; urgent treatment indicated.

Grade V: deaths related to AEs.

9.5.3 Criteria for Determining the Relationship between Adverse Events and the Study Drug

AEs include all unexpected clinical manifestations occurring after the signing of the ICF, which should be reported as AEs, regardless of whether they are related to the study drug, whether they occur in the study drug group, or even whether the drug is administered. Any discomfort reactions or abnormal changes in objective laboratory test indicators complained by the subjects during treatment should be truthfully recorded, and the severity, duration, treatment measures and outcome of the AEs should be noted. The investigator should also comprehensively determine the relationship between the AE and the study drug, and assess the possible relationship between the two according to the five levels of "definitely related, possibly related, unlikely related, not related, not determined". The AEs invloved in the three levels of "definitely related", "possibly related" and "not determined" are listed as adverse drug reactions. The total number of subjects involved in the three levels will be used as the numerator, and the total number of subjects used to evaluate safety will be used as the denominator to calculate the incidence of adverse reactions. The criteria for determination are shown in Table 8:

Table 8 Criteria for Determining the Relationship between Adverse Events and the Study Drug

Grade	Criteria
Definitely related	The event occurs in a reasonable temporal relationship after administration, and is consistent with the known reaction type of suspected study drug; the event is improved after drug withdrawal but reappears after re-administration.
Possibly related	The event occurs in a reasonable temporal relationship after administration, and is not consistent with the known reaction type of suspected study drug; the event may be caused by the subject's clinical condition or other treatments.
Unlikely related	The event does not occur in a reasonable temporal relationship after administration, and is not consistent with the known reaction type of suspected study drug; the event may be caused by the subject's clinical condition or other treatments.
Not related	The event does not occur in a reasonable temporal relationship after administration, and is not consistent with the known reaction type of suspected study drug; the event may be caused by the subject's clinical condition or other treatments; the event is resolved when the disease is improved or the other treatments are discontinued, but reappears when the other treatments are resumed.
Not determined	The event does not occur in a clearly temporal relationship after administration and is similar to the known type of reactions of the study drug, and other concomitant drugs may have also contributed to the corresponding event.

9.5.4 Recording and Reporting of Adverse Events

The investigator should record in detail any adverse events that occur in the subject, including: description of AEs and all relevant symptoms, and their onset time, severity, duration, actions taken and final outcomes (recovered/cured, with or without sequelae, resolved, no change, death, unknown).

In this study, the assessment of drug safety will be performed from the day when the subject signs the ICF to 30 days after the last dose. All AEs (serious and non-serious) should be recorded on the AE page of the CRF and should be reported in accurate medical terms.

Related AEs: follow-up is required until any of the following occurs:

1) AE disappears or improves to baseline;

2) AE is re-evaluated as not related to the study drug (pyrotinib and/or docetaxel);

3) Death;

4) The subject starts a new anti-tumor treatment regimen;

5) The investigator confirms that no further improvement is expected and that the patient's condition is stable;

6) No more collection of clinical data or final database lock.

Unrelated AEs: follow-up is required until any of the following occurs:

1) AE disappears or improves to baseline;

2) The severity is improved to less than Grade 1;

3) Death;

4) The subject starts a new anti-tumor treatment regimen;

5) The investigator confirms that no further improvement is expected;

6) No more collection of clinical data or final database lock.

9.6 Serious Adverse Events (SAEs)

9.6.1 Definition of Serious Adverse Events

Serious adverse event (SAE) is an event that occurs during the clinical trial that requires hospitalization or prolonged hospitalization, leads to disability or incapacity, is life-threatening or fatal, or results in congenital malformations. AEs that meet one or more of the following criteria are SAEs:

- Leading to death;
- Life-threatening (defined as the risk of immediate death of the subject at the time of the event);
- Requiring hospitalization or prolonged hospitalization;
- Resulting in permanent or severe disability/incapacity;
- Resulting in congenital anomalies or birth defects;
- Medically significant events: these AEs may not result in death, be life-threatening, or require hospitalization, but may jeopardize the subject and require medical or surgical intervention to prevent any of the above outcomes based upon appropriate medical judgment.

The investigator should promptly report all SAEs, including their clinical diagnoses, treatments

and outcomes, and follow-up should be performed until it returns to normal, is relieved or stable. In addition, record in detail in the original medical record and eCRF (including the AE report form, and fill in the death record form if it is a death record), and fill in the SAE report form.

9.6.2 Potential Drug-induced Liver Injury

Drug-induced liver injury will be considered if ALT and/or AST levels are abnormal and total bilirubin levels are abnormally increased, the following conditions are met and there are no other causes of liver injury, as shown in Table 9. Such conditions should always be considered as medically significant events.

Table 9 Criteria for Evaluation of Potential Drug-induced Liver Injury

Baseline	Normal (AST/ALT and total bilirubin)		Abnormal (AST/ALT/total bilirubin)	
Treatment Period	ALT≥3×ULN	AST≥3×ULN	ALT or AST ≥ 2 × baseline and ≥ 3 x ULN	AST or ALT ≥ 8 × ULN
	Meet one of the above conditions, with total bilirubin ≥ 2 × ULN and alkaline phosphatase ≤ 2 × ULN and no hemolysis		Meet one of the above conditions, and with total bilirubin ≥ 3 × ULN or increase ≥ 1 × ULN	

Subjects should return to the study site for assessment as soon as possible (preferably within 48 hours) after awareness of the abnormal results. Assessments should include laboratory test, detailed medical history, and physical assessment, and the possibility of liver tumor (primary or secondary) should be considered.

In addition to repeated testing of ALT and AST, laboratory test items should also include albumin, total bilirubin, direct bilirubin and indirect bilirubin, gamma glutamyltransferase, prothrombin time (PT)/international normalized ratio (INR), and alkaline phosphatase. Further tests may include testing for acute hepatitis A, B, C, and E and imaging of liver (such as biliary tract).

Detailed medical history should include alcohol use, acetaminophen, soft drugs, supplements, family's medical history, occupational exposure, sexual activity, travel history, contact with jaundiced patients, surgery, blood transfusion, liver disease, or allergic disease history, etc. If repeated testing of the above items confirms that the definition of the laboratory criteria described above is met, the possibility of potential drug-induced liver injury should be considered in the absence of other causes of abnormal liver function, without waiting for the results of all etiological tests of liver function. Such cases of potential drug-induced liver injury should be reported as SAEs.

9.6.3 Disease Progression

Disease progression (including symptoms and signs of progression) should not be reported as

an SAE, but death due to disease progression within the study or safety reporting period, i.e., within 28 days (including 28 days) after the last dose of study drug, should be reported as an SAE. Hospitalization for symptoms and signs of disease progression should not be reported as an SAE. During the study or safety reporting period, if the final outcome of cancer is death, the event leading to death must be reported as an SAE.

9.6.4 Hospitalization

In this clinical study, the AEs that lead to hospitalization or prolonged hospitalization should be considered as SAEs. Any first hospitalization to a medical facility (even if less than 24 hours) meets this criterion.

The following hospitalizations are not considered SAEs:

- Admission to rehabilitation facilities
- Admission to nursing homes
- Admission to routine emergency room
- Day surgery (such as outpatient/day/ambulatory surgery). Hospitalization or prolonged hospitalization unrelated to an AE is not an SAE. For example:
- Hospitalization for a pre-existing disease, no worsening or new AEs (e.g., hospitalization for laboratory abnormalities that occur prior to the study and still persist);
- Hospitalization due to administrative reasons (e.g., annual routine physical examination);
- On-study hospitalization as specified in the protocol (e.g., protocol-specified procedures);
- Elective hospitalization unrelated to AEs (e.g., elective cosmetic surgery);
- Pre-scheduled treatment or surgery which should be recorded in the protocol and/or in the subject's baseline data;
- Hospitalization only for use of blood products. Diagnostic or therapeutic invasive (e.g., surgery) and non-invasive procedures should not be reported as AEs. However, the condition that results in such procedures should be reported as an AE if it meets the AE criteria. For example, acute appendicitis that occurs during the AE reporting period should be reported as an AE, while the appendectomy thus performed should be recorded as the treatment of the AE.

9.6.5 Serious Adverse Event Reporting System

SAEs should be reported from the time the subject signs the ICF until 28 calendar days (including 28 days) after the last dose of study drug. During the study, in the event of an SAE, regardless of initial or follow-up reporting, the investigator must immediately complete the Serious Adverse Event (SAE) Report Form, with dated signature, notify the CRA, the EC of the study site, the study institution and the sponsor (the study team of Zhejiang Cancer Hospital) within 24 hours after the investigator becomes aware of the SAE (not later than 7 days), and

report and supplement the follow-up information within the next 8 days. Unexpected adverse reactions that are not fatal or life-threatening should be reported as soon as possible after first awareness, but not later than 15 days.

SAEs within 30 days after the last dose are generally not reported unless suspected to be related to the study drug.

SAEs should be recorded in detail, including the symptoms, severity, onset date, treatment time, action taken, concurrent medication, follow-up time and manner, and outcome. If, in the opinion of the investigator, an SAE is not related to the study drug but potentially related to the study conditions (e.g., termination of the prior treatment, or comorbidities during the study), details should be elaborated in the narrative section of the SAE page of the CRF. If the intensity of an SAE or its relationship to the test drug changes, the SAE follow-up report should be submitted to the EC of the study site immediately. All SAEs should be followed up until recovery, resolution to Grade 1 or baseline, or stabilization.

The sponsor will regularly (every 3 months) notify all investigators of SAEs and non-fatal or life-threatening unexpected adverse reactions, and the investigator of each participating institution will submit them to the EC and study institution.

Table 10 Contacts for SAE and SUSAR Reporting

Unit	Contact Department	Reporting Method	Contact Information
Hospitals	EC	Submit in person	
Sponsor	Zhejiang Cancer Hospital	Preferred email, and fax if emailing fails	Email address: huang_jian22@aliyun.com<br>Fax: 0571-88122078

9.7 Pregnancy

If pregnancy is considered an SAE, the CFDA Serious Adverse Event Report Form needs to be completed and submitted in accordance with the time limits and requirements of the SAE.

10 Retrospective Bioinformatic Analysis

This study will perform a retrospective bioinformatic analysis of the subjects, and pre-treatment biopsy specimens (7 slides) will be used to explore the association of biomarkers with the test product (pyrotinib) and to explore the association of biomarker changes with the mechanism of resistance. In addition, tumor samples collected will be used to assess potential predictive candidate biomarkers, including molecular alterations related to HER2 signaling; to assess the mechanism of action of therapy, breast cancer biology, or both; to assess the relationship of potential biomarkers to treatment safety; or to improve diagnostic accuracy.

Biomarkers may be phosphatase, phosphatase gene (PTEN), phosphatidylinositol kinase (PI3K), HER family receptor proteins, or RNA expression messengers, etc. The final marker testing will be performed according to the test items of the testing company (Beijing Pu Kang

Rui Ren Medical Laboratory Co., Ltd.).

OncoClear® is a test product targeting 561 genes related to cancer mechanism for precise cancer treatment launched by Beijing Pu Kang Rui Ren Medical Laboratory Co., Ltd. This product utilizes probe capture combined with high-throughput sequencing technology to analyze all exon regions of 561 genes, hotspot mutation regions (intron or promoter regions) of some genes, biomarkers such as tumor mutation burden (TMB) and microsatellite instability (MSI), and sites related to the use of chemotherapeutic drugs to comprehensively assess the suitability of targeted therapy, immunotherapy, endocrine therapy and chemotherapeutic drugs, thus providing clinicians with comprehensive tumor treatment options and assisting clinicians to achieve precise treatment.

11 Study Management

11.1 Ethics and Informed Consent

11.1.1 Ethics

This clinical study must be conducted in accordance with the Declaration of Helsinki (2008), Good Clinical Practice (GCP) promulgated by NMAP (formerly CFDA) (2020 Edition), and relevant regulations. Approval must be obtained from the EC of the responsible study site prior to initiation of the study. During the clinical study, any modifications to this protocol should be reported to the EC and filed. The investigator has the responsibility to submit interim reports of the study regularly as required by the EC. When the study ends, the EC should be informed.

11.1.2 Informed Consent

Subjects must give informed consent to participate in this study prior to receiving study treatment in order to protect their legal rights and interests. It is the responsibility of the investigator to provide the subject or his/her designated representative with a complete and comprehensive description of the objectives of the study, the effects of the drug, possible toxic and side effects, and possible risks, and to inform the subjects of their rights, risks and benefits. Conversation is a very important informed consent process. If the subject and his/her legal representative are illiterate, the informed consent process should be attended by a witness, who will sign the informed consent form after the subject or his/her legal representative gives oral consent, and the signature of the witness should be on the same day as the subject's signature. The informed consent form should indicate the version number and version date.

11.2 Study Drug Management

The management, dispensing and return of study drugs in this study are the responsibility of a special person. The investigator must ensure that all study drugs are used only in the subjects participating in the clinical study, the dosage and usage will comply with the protocol, the

unused drugs will be returned to the study site, and the study drugs will not be transferred to any non-clinical study participants.

The CRA is responsible for monitoring the supply, use, and storage of the study drugs and disposal of unused study drugs.

11.3 Amendments to the Protocol

The protocol may not be modified by anyone other than the Leading Study Unit. Any necessary changes to the protocol should be made in the form of protocol amendments and submitted to the EC for approval or filing after obtaining the signature and consent of the investigator, and details of previous amendments should be described in the protocol.

11.4 Monitoring

The Leading Study Unit shall appoint persons with appropriate medical, pharmaceutical, or related professional qualifications, who have received necessary training and are familiar with the GCP and relevant laws and regulations, as CRA for this clinical study to monitor and report the conduct of the study and verify the data, in order to ensure that the rights and interests of subjects are safeguarded in the clinical study, the data recorded and reported are accurate and complete, and the study complies with the approved protocol, GCP and relevant laws and regulations.

The monitor will supervise the AEs and SAEs to verify that all AEs are accurately, reliably and timely recorded and reported in the clinical study.

11.5 Quality Control and Assurance

• The clinical study institution must be a drug clinical study site with clinical study conditions identified by NMAP (formerly CFDA);

• The study personnel must be physicians trained in clinical studies and work under the guidance of senior professionals;

• Before a study, the clinical ward must meet the requirements of standardization and ensure that rescue equipment is complete;

• The subject should be given medication by a professional nursing staff to understand the drug intake in detail and ensure the subject's compliance;

• Each site must strictly follow the protocol and truthfully complete the eCRF;

• The monitor should follow standard operating procedures to supervise the conduct of the clinical study, confirm that all data records and reports are correct and complete and all eCRFs are completed correctly and consistent with the original data, and ensure that the study is conducted in accordance with the clinical study protocol;

• In the event of an SAE, the monitor should promptly notify the study site, and temporarily discontinue the study if necessary;

• All sites participating in the study should be audited by drug regulatory authorities, and it is particularly important that the investigator and his/her relevant personnel should provide convenience and time for monitoring and audit.

11.6 Data Management

11.6.1 Data Collection

The eCRFs will be used for the collection of study data in this study; the monitor will train designated study facility personnel on the EDC system. Study facility personnel cannot log into the EDC system before receiving the training. The investigator or dedicated data entry personnel (CRC) should enter the data into the EDC system in accordance with the requirements of the visit process and the eCRF completion guidelines. The system edit check procedure will check the completeness and logic of the clinical study data entered into the EDC system, and generate error prompts on the data in question, allowing the investigator or CRC to modify or explain the data in question. After database lock, the investigator will receive a copy of CD-ROM or documentation of the patient's data for archiving at the study site.

11.6.2 Data Management and Quality Control

In order to ensure the authenticity and reliability of clinical study data and improve the quality of clinical data, the CRA will review the integrity, consistency and accuracy of the study data in the clinical database in accordance with the standard operating procedures during the conduct of the study, and guide the study stie personnel to supplement or correct the data in question as necessary. The CRA or data administrator will query the data in question to the investigator or CRC by an Electronic Query Form. The investigator or CRC must respond to the query and make corrections or explanations to the questioned data, and queries may be issued for multiple times if necessary until the data in question are resolved. At the end of the study, data administrator and medical personnel will perform final quality control on all data in the database, summarize all protocol deviations and violations during the study, and convene a data verification meeting. After the data in the database meets the quality requirements, the database will be locked, and the data administrator will export the data for analysis by the statistical department.

11.6.3 Review of Data and Monitoring of the Study Site

Prior to study initiation, the CRA will introduce the protocol and eCRFs (study Part 2) at the initial visit to the study site or at the investigator meeting. During the study, the CRA will regularly visit the study site and check the completeness of patient records and the accuracy of eCRF, compliance with the protocol and Good Clinical Practice, and progress of enrollment, and ensure that the study drugs are stored, distributed and counted in accordance with the requirements. Key study personnel must be able to assist the CRA during these visits. The

investigator must maintain the original documents of each patient participating in the study, including medical records and visit records (inpatient or outpatient medical records), which include demographic indicators and medical information, laboratory data, electrocardiograms, and results of any other tests or evaluations. All information on the eCRF must be derived from the original document in the patient's file. The investigator must also keep the ICF signed by the patient.

The investigator must confirm that all relevant original documents can be monitored to confirm that they are consistent with the eCRF. The monitoring criteria requires 100% monitoring of the obtained ICF, compliance with the inclusion/exclusion criteria, the recording of SAEs, and the data required for the evaluation of all primary and safety variables. Additional check for consistency of the original data with the eCRF will be performed in accordance with the monitoring plan in the study. Any information on the patient identification in the original documents will not be disclosed.

11.7 Protocol Violations

All requirements specified in the protocol must be strictly followed. Any intentional or unintentional behavior that deviates from or violates the protocol or GCP principles should be classified as a protocol deviation or violation. Any deviation from protocol found by the CRA during the monitoring process should be recorded by the investigator or CRA with details of time of discovery, time of occurrence, process, cause, and corresponding measures taken. The record should be signed by the investigator, and the EC should be notified.

11.8 Data Storage

To ensure that the National Medical Products Administration can conduct evaluation and supervision of the clinical study, the investigator should agree to maintain all study data, including original inpatient records, ICF, CRF, detailed records of drug dispensing, etc. and the study data should be retained by the study site until 5 years after the end of the clinical study. All data of this clinical study are the property of the Leading Study Unit, and no one may provide it to a third party in any form without the written consent of the Leading Study Unit, except as required by the China Food and Drug Administration.

11.9 Publication of Study Results

All study-related articles and reports may be published only with the consent of the investigator.

12 Data Analysis and Statistical Methods

The detailed methods for summarization and statistical analysis of the data collected for this study will be included in the Statistical Analysis Plan (SAP), which will be finalized and filed by the Principal Investigator. If a change is made to the protocol that will have a significant

impact on the SAP as judged by the principal investigator, the SAP should also be revised to be consistent with the protocol.

12.1 Sample Size Calculation

A Simon two-stage design will be used for this study. The objective response rate of trastuzumab combined with docetaxel is 60% based on literature reports and data from previous clinical studies, and an objective response rate of 75% is expected for pyrotinib combined with docetaxel in this study. If the significance level $\alpha = 0.05$ (one-sided) and $1-\beta = 0.80$, the PASS 15.0 Two-Stage Phase 2 Clinical Trial module can be used to obtain:

The study will be conducted in 2 parts. In Part I, 27 evaluable subjects are required to be enrolled. If ≤ 17 subjects do not achieve CR or PR, the study will be prematurely discontinued, and the objective response rate in the test group does not reach the treatment level of the control group; if > 17 subjects achieve CR or PR, the enrollment of subjects in Part 2 will continue. Finally, 67 evaluable subjects need to be included in this study. Of the 67 evaluable subjects, if > 46 subjects achieve CR or PR, the study will be considered to achieve the expected endpoint. Considering that a dropout rate of 15%, at least 79 subjects should be enrolled in the test group.

12.2. Study Hypotheses

This study will test the following hypotheses:

H_0: ORR < 60% in the test group

H_1: ORR ≥ 75% in the test group

12.3 Data Analysis Sets

The analysis populations for this study include the Full Analysis Set (FAS), the Per Protocol Set (PPS), and the Safety Set (SS).

Full Analysis Set: analysis set determined according to the intention-to-treat analysis (ITT) principle. All enrolled subjects with at least one documented dose (pyrotinib combined with docetaxel) and who have received at least one post-treatment efficacy assessment will be included in this analysis set.

Per Protocol Set: a subset of the Full Analysis Set. All enrolled subjects who have at least one medication record and do not experience major protocol violations that are considered to affect the efficacy analysis will be included in this analysis set.

Safety Set: all enrolled subjects who have taken at least one dose of the study drug and have at least one post-dose safety record will be included in the Safety Set. The Safety Set is mainly used for safety analysis.

Baseline analysis in this study is mainly for the FAS, and the efficacy analysis is mainly for the FAS and PPS, of which the FAS is the primary analysis set, and the safety analysis is mainly for the SS.

12.4 Statistical Analysis Plan

This study will apply SAS version 9.3 and above for statistical analysis.

12.4.1 Statistical Description

Unless otherwise specified, the study data will be summarized descriptively in accordance with the following general principles.

Continuous data will be summarized descriptively using statistics including mean, standard deviation (SD), median, minimum, and maximum. Binary categorical data will be summarized descriptively using statistics including frequency (n) and percentage (%), with 95% confidence intervals for overall percentages, if necessary. The Kaplan-Meier method will be used to estimate the survival function, plot the survival curve, and calculate the median survival time and its 95% confidence interval representing the overall survival time.

12.4.2 Efficacy Analysis

The primary endpoint of this study is ORR, and ORR and its 95% confidence interval will be calculated in the test group.

The secondary endpoints PFS, OS and DoR will be plotted by Kaplan-Meier method, and median survival with 95% confidence interval will be presented.

The secondary endpoint CBR and its 95% confidence interval will be calculated in the test group.

12.4.3 Safety Analysis

Safety analysis will be limited to descriptive statistical summaries, mainly including, but not limited to, the following: subject disposition and analysis population; basic characteristics of subjects (including sociodemographic information, past medical history and prior medication history); subject discontinuation from the study; summary of AEs (all-cause and treatment-related); incidence and severity of AEs (all-cause and treatment-related); summary of SAE details; relationship analysis of AEs; vital signs, physical examination, laboratory data and change from baseline, and the number and percentage of subjects who change from "normal to abnormal" or have "worsening of abnormalities" after the study.

13 Dropouts

All subjects who have completed the ICF and have been eligible to enter the study have the right to withdraw from the clinical study at any time. Whenever and for whatever reason, subjects who have not completed 1 cycle of the clinical study and cannot be evaluated for safety and efficacy will be considered dropouts (those who leave the study due to disease progression after enrollment and have documented medical evidence are not considered dropouts). When a subject drops out, the investigator must fill the reason for dropout in the eCRF, complete the

assessments that can be completed, and carefully complete the last visit record in the eCRF. If a subject drops out due to an adverse reaction and the reaction is finally assessed to be related to the study drug in follow-up visit, it should be recorded in the eCRF and notified to the investigator. Subjects who withdraw from the study after screening without obtaining a drug number will not be considered dropouts. Subjects who have completed a full cycle with detailed records will be included in the statistical analysis for safety evaluation. Subjects who have withdrawn from the study are not allowed to re-enter the study and their numbers could not be reused.

14 References

[1] Freudenberg JA, et al. The role of HER2 in early breast cancer metastasis and the origins of resistance to HER2-targeted therapies. Exp Mol Pathol. 2009 Aug;87(1):1-11.

[2] Marty M, et al. Randomized phase II trial of the efficacy and safety of trastuzumab combined with docetaxel in patients with human epidermal growth factor receptor 2-positive metastatic breast cancer administered as first-line treatment: the M77001 study group. J Clin Oncol. 2005;23 (19) :4265-74

[3] Marty M, et al. Randomized phase II trial of the efficacy and safety of trastuzumab combined with docetaxel in patients with human epidermal growth factor receptor 2-positive metastatic breast cancer administered as first-line treatment: the M77001 study group. J Clin Oncol. 2005;23 (19) :4265-74.

[4] HE Jie, CHEN Wanqing. Published in 2017 China Cancer Registry Annual Report [M] .Beijing: People's Medical Publishing House, 2018.
