# Supplementary material for: Pyrotinib plus docetaxel as first-line treatment for HER2-positive metastatic breast cancer: the PANDORA phase II trial
Source: Nat Commun. 2023 Dec 14;14:8314. doi: 10.1038/s41467-023-44140-y (PMC10721794; doi:10.1038/s41467-023-44140-y)
Supplement: Supplementary file 3 — Reporting Summary [file 41467_2023_44140_MOESM3_ESM.pdf]

nature portfolio

Corresponding author(s): Xiaojia Wang, Zhanhong Chen

Last updated by author(s): Oct 7, 2023

Reporting Summary

Nature Portfolio wishes to improve the reproducibility of the work that we publish. This form provides structure for consistency and transparency in reporting. For further information on Nature Portfolio policies, see our Editorial Policies and the Editorial Policy Checklist.

Statistics

For all statistical analyses, confirm that the following items are present in the figure legend, table legend, main text, or Methods section.

n/a	Confirmed	
☐	☒	The exact sample size (*n*) for each experimental group/condition, given as a discrete number and unit of measurement
☐	☒	A statement on whether measurements were taken from distinct samples or whether the same sample was measured repeatedly
☐	☒	The statistical test(s) used AND whether they are one- or two-sided<br>*Only common tests should be described solely by name; describe more complex techniques in the Methods section.*
☐	☒	A description of all covariates tested
☐	☒	A description of any assumptions or corrections, such as tests of normality and adjustment for multiple comparisons
☐	☒	A full description of the statistical parameters including central tendency (e.g. means) or other basic estimates (e.g. regression coefficient) AND variation (e.g. standard deviation) or associated estimates of uncertainty (e.g. confidence intervals)
☐	☒	For null hypothesis testing, the test statistic (e.g. *F*, *t*, *r*) with confidence intervals, effect sizes, degrees of freedom and *P* value noted<br>*Give P values as exact values whenever suitable.*
☒	☐	For Bayesian analysis, information on the choice of priors and Markov chain Monte Carlo settings
☒	☐	For hierarchical and complex designs, identification of the appropriate level for tests and full reporting of outcomes
☐	☒	Estimates of effect sizes (e.g. Cohen's *d*, Pearson's *r*), indicating how they were calculated

Our web collection on statistics for biologists contains articles on many of the points above.

Software and code

Policy information about availability of computer code

Data collection	Data collection was performed in Microsoft Office Excel 2019.
Data analysis	All statistical analyses were conducted using SAS version 9.3, R package named survival (v3.3.1) and R package named ggplot2 (v3.3.6). The sample size was calculated using NCSS&PASS version 15.0.

For manuscripts utilizing custom algorithms or software that are central to the research but not yet described in published literature, software must be made available to editors and reviewers. We strongly encourage code deposition in a community repository (e.g. GitHub). See the Nature Portfolio guidelines for submitting code & software for further information.

Data

Policy information about availability of data

All manuscripts must include a data availability statement. This statement should provide the following information, where applicable:

- Accession codes, unique identifiers, or web links for publicly available datasets
- A description of any restrictions on data availability
- For clinical datasets or third party data, please ensure that the statement adheres to our policy

The trial protocol is provided in the Supplementary Information. Deidentified clinical data for individual patients supporting the results of this manuscript are included within the manuscript and its additional files. To ensure patient privacy, further deidentified data are not publicly available, but can be accessed upon a scientifically justified request to the corresponding author, Xiaojia Wang (wxiaojia0803@163.com), for up to ten years after this paper's publication. The corresponding author will review all data requests to ensure its scientific use, and upon approval, de-identified patient data will be shared within three months. The

raw sequence data reported in this paper have been deposited in the Genome Sequence Archive in National Genomics Data Center, China National Center for Bioinformation/Beijing Institute of Genomics, Chinese Academy of Sciences (GSA-Human: HRA005624) that are publicly accessible at https://ngdc.cncb.ac.cn/gsa-human. Source data are provided with this paper. The remaining data are available within the Article, Supplementary Information or Source Data file.

Research involving human participants, their data, or biological material

Policy information about studies with human participants or human data. See also policy information about sex, gender (identity/presentation), and sexual orientation and race, ethnicity and racism.

Reporting on sex and gender	Our study exclusively involves female patients due to its focus on breast cancer.
Reporting on race, ethnicity, or other socially relevant groupings	Our study only includes Chinese patients.
Population characteristics	The median age of the patients was 52 years, ranging from 28 to 70 years. Out of all participants, 33 (41.8%) had previously received taxane, and 24 (30.4%) had received trastuzumab in the (neo)adjuvant setting.
Recruitment	All woman with HER2-positive metastatic breast cancer who had not received HER2 blockade or chemotherapy for metastatic disease between June 2019 and June 2021 were included. Eligible patients met the following criteria: 1) histologically confirmed MBC and candidates for chemotherapy; 2) HER2-positive, defined as immunohistochemistry (IHC) staining 3+ or IHC staining 2+ with fluorescence in situ hybridization positive; 3) aged between 18 and 70 years old; 4) an Eastern Cooperative Oncology Group performance status (ECOG PS) of 0-1; 5) at least one measurable disease according to Response Evaluation Criteria in Solid Tumors (RECIST) version 1.1; 6) may have received hormonal regimens in the metastatic setting; 7) (neo)adjuvant trastuzumab or taxane treatment was permitted, with a disease-free interval of more than 12 months from completion of the taxane and more than 6 months from completion of the trastuzumab. Patients who had previously received anti-HER2 TKI were excluded, as were those with CNS metastasis. Detailed inclusion and exclusion criteria can be found in the protocol.
Ethics oversight	The study was conducted in accordance with the Declaration of Helsinki and Good Clinical Practice principles. The protocol was approved by the ethics committee of Zhejiang Cancer Hospital, Sun Yat-sen University Cancer Center, Harbin Medical University Cancer Hospital, Nanchang People's Hospital, Fujian Cancer Hospital, The First Affiliated Hospital, Zhejiang University School of Medicine, The Second Affiliated Hospital, Zhejiang University School of Medicine, Sir Run Run Shaw Hospital, Zhejiang University School of Medicine, Beijing Cancer Hospital and The Second Affiliated Hospital of Dalian Medical University. All patients provided written informed consent before any procedure.

Note that full information on the approval of the study protocol must also be provided in the manuscript.

Field-specific reporting

Please select the one below that is the best fit for your research. If you are not sure, read the appropriate sections before making your selection.

☒ Life sciences ☐ Behavioural & social sciences ☐ Ecological, evolutionary & environmental sciences

For a reference copy of the document with all sections, see nature.com/documents/nr-reporting-summary-flat.pdf

Life sciences study design

All studies must disclose on these points even when the disclosure is negative.

Sample size	The study was conducted using a two-stage Simon design. Based on previous data of trastuzumab and docetaxel as a first-line treatment in HER2-positive MBC, the null hypothesis for the ORR was set at 60% (Marty, M. et al. J Clin Oncol, 2005)., while the alternative hypothesis was an ORR of 75%. With a one-sided α of 0.05 and a power of 0.80, the first stage required 27 evaluable patients. If more than 17 patients achieved responses in the first stage, an additional 40 evaluable patients would be enrolled in the second stage. If more than 46 out of 67 patients achieved responses in total, the results were considered positive. Considering a dropout rate of 15%, a total of 79 patients were required for this study.
Data exclusions	No data were excluded for the analysis
Replication	Due to limited sample of tumor biopsy, we only performed biomarker analysis once.
Randomization	This is a single-arm study, so randomization was not performed.
Blinding	This is a single-arm study, so blinding is not application.

Reporting for specific materials, systems and methods

We require information from authors about some types of materials, experimental systems and methods used in many studies. Here, indicate whether each material, system or method listed is relevant to your study. If you are not sure if a list item applies to your research, read the appropriate section before selecting a response.

Materials & experimental systems

n/a	Involved in the study	
☒	☐	Antibodies
☒	☐	Eukaryotic cell lines
☒	☐	Palaeontology and archaeology
☒	☐	Animals and other organisms
☐	☒	Clinical data
☒	☐	Dual use research of concern
☒	☐	Plants

Methods

n/a	Involved in the study	
☒	☐	ChIP-seq
☒	☐	Flow cytometry
☒	☐	MRI-based neuroimaging

Clinical data

Policy information about clinical studies
All manuscripts should comply with the ICMJEguidelines for publication of clinical research and a completedCONSORT checklist must be included with all submissions.

Clinical trial registration	The study was registered on clinical trial.gov with the registration number NCT03876587.
Study protocol	The trial protocol is available in the Supplementary file.
Data collection	PANDORA is a multicenter, single-arm phase II trial with a Simon two-stage design, involving woman with HER2-positive MBC who had not received HER2 blockade or chemotherapy for metastatic disease between June 12, 2019 and June 18, 2021. The trial was conducted at 10 centers in China (Zhejiang Cancer Hospital, Sun Yat-sen University Cancer Center, Harbin Medical University Cancer Hospital, Nanchang People's Hospital, Fujian Cancer Hospital, The First Affiliated Hospital, Zhejiang University School of Medicine, The Second Affiliated Hospital, Zhejiang University School of Medicine, Sir Run Run Shaw Hospital, Zhejiang University School of Medicine, Beijing Cancer Hospital and The Second Affiliated Hospital of Dalian Medical University).
Outcomes	The primary endpoint of this study was the objective response rate (ORR), defined as the proportion of patients who achieved a confirmed complete response (CR) or partial response (PR). Secondary endpoints included overall survival (OS), which was defined as the time from obtaining informed consent to death from any cause, and progression-free survival (PFS), defined as the time from obtaining informed consent to disease progression or death from any cause, whichever came first. Other secondary endpoints were the clinical benefit rate (CBR), defined as the proportion of patients with confirmed CR, PR, or stable disease (SD) lasting for at least 24 weeks, and the duration of response (DoR), defined as the time from the first documented response to disease progression for patients with confirmed CR and PR, and the time from treatment initiation to disease progression for patients with SD. Safety profiles were also assessed as a secondary endpoint. Additionally, exploratory endpoints included the time to response (TTR), which was calculated as the time from treatment initiation to the first documented response, and the incidence of CNS metastasis as the first site of disease progression.
